# Comparative analysis of antioxidant activity and structural changes of *Gastrodiae Rhizoma* polysaccharides between sulfur-fumigation and nonsulfur-fumigation

**DOI:** 10.3389/fnut.2024.1477689

**Published:** 2024-12-04

**Authors:** Chunyan Dai, Dan Zhao, Wenping Zhang, Lanping Guo, Chuanzhi Kang, Zhuowen Chen, Xiuming Cui, Tao Zhou, Chengxiao Wang, Tingting Xu, Ye Yang

**Affiliations:** ^1^School of Life Science and Technology, Kunming University of Science and Technology, Kunming, China; ^2^Key Laboratory of Sustainable Utilization of Panax Notoginseng Resources of Yunnan Province, Kunming, China; ^3^Postdoctoral Fellow, Mobile Station of Environmental Science and Engineering, Kunming University of Science and Technology, Kunming, China; ^4^Guizhou University of Traditional Chinese Medicine, Guiyang, China; ^5^Chinese Medica Resources Center, China Academy of Chinese Medicinal Sciences, Beijing, China; ^6^Kunming Medical University Haiyuan College, Kunming, China

**Keywords:** *Gastrodiae Rhizoma*, *Gastrodia elata* polysaccharides, sulfur fumigation, structural characterization, antioxidant activity, glycosidic bond

## Abstract

**Introduction:**

*Gastrodiae Rhizoma* (referred to Tianma in Chinese), the dried tuber of *Gastrodia elata* Bl. (Orchidaceae), is utilized as a medicine-food homology product. Sulfur fumigation is commonly employed in the processing of *Gastrodiae Rhizoma* (GR). Polysaccharides are a crucial active substance produced in GR, yet the impacts of sulfur fumigation on them remain unelucidated.

**Methods:**

This study aimed to optimize the hot water extraction conditions of polysaccharides from sulfur-fumigated GR (SGCPs) and nonsulfur-fumigated GR (NGCPs). The research explored the effects of sulfur fumigation on the structure and antioxidant activity of GR polysaccharides.

**Results and discussion:**

The results showed that the optimal extraction conditions for SGCPs and NGCPs were 67°C for 31 min with a liquid-to-material ratio of 15 mL/g and 64°C for 32 min with a liquid-to-material ratio of 17 mL/g, respectively. Compared with NGCPs, SGCPs exhibited significantly reduced DPPH radical, hydroxyl radical, ABTS^+^ radical scavenging activity, and Fe^2+^ chelating ability. Moreover, both NGCPs and SGCPs offered significant protective effects against H_2_O_2_^−^induced oxidative damage in RAW264.7 cells, but the protective effect of SGCPs was significantly lower than that of NGCPs. NMR analyses revealed that the main chain connections of SGCP3 and NGCP3 were both →4)-*α*-D-Glcp-(1→, and sulfur fumigation increased the number of repeating unit structures →4)-D-Glcp-(1 → in GR polysaccharides. SGCP3 and NGCP3 had the same monosaccharides composition but different molar ratios, with molecular weights of 727,650 and 39,991 Da, respectively. In general, sulfur fumigation reduced the antioxidant activities of GR polysaccharides by altering their structure and composition.

## 1 Introduction

*Gastrodiae Rhizoma* (Tianma) (GR) is a rhizome of *Gastrodia elata* Bl., which belongs to Orchidaceae family, holds significance as both a valuable herb and a significant functional food. In 2019, GR was officially approved to be listed in the catalogue of medicinal and food homologous varieties in China, possessing legal food properties (1). In 2023, GR was also recognized by the Chinese government as a “food and medicine substance” with a huge market value and development potential. GR, as a variety with medicine-food homology, containing active substances such as *Gastrodia Rhizoma* polysaccharide, gastrodin and p-hydroxybenzyl alcohol. Among them, the polysaccharide content of GR can reach up to 25.94–31.31% (2). These polysaccharides exert antidepressant, antioxidative, cardioprotective, and anti-dizziness effects. They also improve memory, delay aging, lower blood pressure, protect cardiovascular and cerebral blood vessels, enhance immunity, scavenge free radicals, etc. (3, 4). As a result, it presents promising prospects in the fields of food, health care, and cosmetic.

Sulfur fumigation is commonly used while processing Chinese herbal medicines. In 1900, the county record of Wenxian County in Henan Province, China, documented that local farmers fumigated yam with sulfur (5). Chinese herbal medicines have a complex and diverse composition. During sulfur fumigation, sulfur and water generated by sulfurous acid react with compounds in Chinese herbal medicines, thereby changing the content of the compounds present or leading to the production of new compounds, which thus modifies the medicinal composition of the herbs and even their safety. For example, sulfur fumigation decreased the analgesic effect of *Angelica dahurica* Benth. (6). Zhan et al. (7) revealed that the aqueous extract of sulfur-fumigated *A. sinensis* (Oliv.) Diels (S-ASR) exerted a strong inhibitory effect on the viability of MCF-7 cells, whereas that of nonsulfur-fumigated *A. sinensis* (Oliv.) Diels (ASR) had no inhibitory effect on cell viability. ASR was superior to S-ASR in the inhibition of platelet aggregation, induction of NO production by HUVEC cells, and elevation of estrogenic activity in MCF-7 mammary gland cells. In studying the effect of sulfur-fumigated *Panax ginseng* C. A. Mey. on immune function in mice, Guo et al. (8) found that *P. ginseng* C. A. Mey. containing a high SO_2_ dose (484.5 μg/g) significantly reduced the immunomodulatory and antioxidant effects. According to Fang et al. (9), nonsulfur-fumigated *P. ginseng* C. A. Mey. polysaccharides had a higher extraction rate, a wider molecular weight distribution, lower branching, higher linearity, and stronger immunomodulatory activity than sulfur-fumigated polysaccharides in immunocompromised mice.

Sulfur fumigation is also a common processing method used for GR. Sulfur-fumigated GR has a white and bright appearance and can prevent rotting mold (10). In our previous study, sulfur fumigation reduced the content of aspalathin, balisenoside, balisenoside B, and balisenoside C (11), and accelerated nutrient decomposition, thereby reducing the edible taste of GR (12). The research hotspots for sulfur-fumigated GR have only focused on the effect of the small molecule content of medicinal substances represented by aspalathin. They have neglected the effect of active biomacromolecules such as polysaccharides. Therefore, exploring the effects of sulfur fumigation on the structure and activity of GR polysaccharides and analyzing the mechanism underlying the changes in its bioactivity is of great significance in ensuring the safety of drug use.

In this study, the extraction process of GR polysaccharides (SGCPs and NGCPs) was optimized, and polysaccharides with good antioxidant activity were selected for purification and structural characterization. The research results can offer a theoretical basis and data support for the scientific processing of GR and the development of GR polysaccharide products.

## 2 Materials and methods

### 2.1 Sulfur fumigation of GR

Fresh GR (Grade II, 2 years old, weighing 100–200 g per GR) was harvested in December 2021 from the GR planting base in Xiaocaoba Town, Yiliang County, Zhaotong City, Yunnan Province, China (E104°15′, N27°46′). The GR was identified by Researcher Cui Xiuming from Kunming University of Science and Technology as the dried tuber of *Gastrodia elata* Bl., a plant belonging to Orchidaceae family. The preparation of dried GR samples in this study was conducted as described in our previous study (12). Briefly, the fresh GR was washed with tap water and steamed in a steamer for approximately 20 min until no white heart was observed in the cross-section, removed, and cooled to room temperature (Supplementary Figure S1). Then, the steamed GR was fumigated for 90 min in a 1:20 ratio of sulfur to GR weight and dried in a 50°C oven. Meanwhile, nonsulfur-fumigated GR after steaming was also dried directly in a 50°C oven as a control. After GR was dried, it was crushed and stored at room temperature for later use (Supplementary Figure S1). All samples were purchased from the same batch, and each treatment was subjected to no less than three sets of repeated operations.

### 2.2 Extraction of GR crude polysaccharides

The sulfur-fumigated and nonsulfur-fumigated GR samples were crushed and sieved (60-mesh). Crude polysaccharides were extracted from the GR by using the hot water immersion method. The single-factor extraction experiments for GR crude polysaccharides were designed based on the research findings of Yang et al. (13) and Wang et al. (14). GR (10 g) was weighed and extracted under the conditions of liquid to material ratio (5, 10, 20, 30, 40, and 50 mL/g), extraction time (15, 30, 60, 90, 120, and 150 min) and extraction temperature (30, 40, 50, 60, 70, and 80°C). The extract was centrifuged at 25°C, 4,000 rpm/min, for 5 min. The filtrate was measured in a 250 mL volumetric flask. The content of polysaccharide was determined through the phenol-sulfuric acid method by using D-Glucose as the reference standard. According to the method of Yang et al. (13), the crude polysaccharide extraction rate was calculated using the following formula:

$$\mathrm{Crude polysaccharide extraction rate}\;\left(\%\right)=\frac{C\times V_{T}\times n}{W\times V_{S}}$$

Where: *C* is the concentration of crude polysaccharide, mg/mL; *V_T_* is the total volume of the extract, mL; *n* is the number of dilutions; *W* is the mass of the sample, g; and *V_S_* is the volume of the sample taken for the determination, mL.

Based on the initial screening of extraction factors in a one-way test, three factors, namely A (extraction temperature, °C), B (extraction time, min), and C (liquid-to-material ratio, mL/g) were studied to determine their influence on the crude polysaccharide yield. According to the Box–Behnken Design method, three levels were designed, namely A (50, 60, 70), B (15, 30, 45), C (10, 20, 30). The design of each factor and level is shown in Table 1; the whole design consisted of the group of 17 experiments in Table 2.

**Table 1:** Response surface factor level table.

Variant	Unit	Notation	Level		
			−1	0	1
Extraction temperature	°C	A	50	60	70
Extraction time	min	B	15	30	45
Liquid to material ratio	mL/g	C	10	20	30

**Table 2:** Response surface method design and results.

Experiment No.	Extraction temperature: A (°C)	Extraction time: B (min)	Liquid to material ratio: C (mL/g)	GR polysaccharide extraction rate: Y (%)	
				sulfur-fumigated	nonsulfur-fumigated
1	50	45	20	5.24 ± 0.08	13.77 ± 0.96
2	70	15	20	8.09 ± 0.80	14.86 ± 1.36
3	70	45	20	9.54 ± 0.78	17.52 ± 0.30
4	60	15	10	12.12 ± 0.40	22.92 ± 0.36
5	60	30	20	17.23 ± 0.41	26.23 ± 1.07
6	70	30	30	8.32 ± 0.74	15.18 ± 0.91
7	50	15	20	4.29 ± 0.83	10.40 ± 0.51
8	60	30	20	18.59 ± 1.01	28.54 ± 1.42
9	60	30	20	17.31 ± 0.27	26.31 ± 1.34
10	60	15	30	9.42 ± 0.37	19.42 ± 0.63
11	70	30	10	11.11 ± 0.21	18.04 ± 0.30
12	60	45	10	12.77 ± 0.69	24.09 ± 1.85
13	50	30	10	9.43 ± 0.89	23.99 ± 0.35
14	60	45	30	7.81 ± 0.46	17.00 ± 0.27
15	50	30	30	7.30 ± 0.46	17.48 ± 0.40
16	60	30	20	16.01 ± 0.48	27.78 ± 0.53
17	60	30	20	15.31 ± 0.46	27.98 ± 0.84

### 2.3 Isolation and purification of GR polysaccharides

The crude extraction liquid of GR polysaccharides, obtained through optimized extraction processes, was subjected to ethanol precipitation, protein removal using Sevag reagent, and dialysis, ultimately yielding polysaccharides after freeze-drying. Subsequently, the polysaccharides were separated using a DEAE-52 cellulose column (Cytiva, United States, 0–0.5 mol/L NaCl linear gradient elution). The purified fractions were then processed on a Sephadex G-50 dextran gel chromatography column (Shanghai Yuanye Biotechnology Co., Ltd., 0.1 mol/L NaCl elution). The eluent was collected in 10 mL per tube. The elution curve was plotted based on the absorbance at 490 nm (15). Samples were collected based on the elution curve, dialyzed (Mw 3,500 Da), and freeze-dried under vacuum for subsequent experiments. The intuitive purification process was shown in Supplementary Figure S1.

### 2.4 Determination of antioxidant activity

#### 2.4.1 Determination of antioxidant activity

Referring to Ji et al. (16), DPPH, hydroxyl radical, and ABTS^+^ radical scavenging activities, Fe^2+^ chelating capacity, and total reducing power of SGCPs and NGCPs were determined. Vc and EDTA-2Na were selected as positive controls (Specific experimental methods can be found in Supplementary methods).

#### 2.4.2 Antioxidant activity determination at the cellular level

##### 2.4.2.1 Cell culture

Murine macrophage RAW264.7 cells were purchased from the Cell Resource Center of the Chinese Academy of Medical Sciences and Peking Union Medical College (Beijing, China). RAW264.7 cells were cultured in a complete medium containing 10% fetal bovine serum, 1% penicillin streptomycin, and 90% DMEM (5% CO_2_, 37°C).

##### 2.4.2.2 Effect of polysaccharides on RAW264.7 cell viability

According to the method of Zhou et al. (17), RAW264.7 cells were cultured for 24 h. After removing the supernatant, complete culture media with different polysaccharide concentrations (0, 12.5, 25, 50, 100, 200 μg/mL) were added and incubated at 37°C for 24 h. Three sub-wells were established for each concentration. After centrifugation for 10 min (4,000 rpm/min, 25°C) to remove the supernatant, 30 μL MTT (5 mg/mL) was added to each well. After incubating the cells for 4 h at 37°C, the supernatant was removed, 100 μL DMSO solution was added and gently shaken for 10 min. The absorbance at 490 nm was detected and the results were read using microplate reader (Thermo Fisher Varioskan LUX, United States). The results were expressed as percentages.

##### 2.4.2.3 Modeling of oxidative damage

The RAW264.7 cells were cultured for 24 h. After all medium was removed, H_2_O_2_ solution (100 μL/well) was added at concentrations of 0, 0.125, 0.25, 0.5, 1, and 2 mmol/L. The MTT method used was the same as that given in 2.4.2.2. The concentration of H_2_O_2_ that causes significant damage to cells without causing excessive cell death was identified by setting the threshold of cell viability at 50%.

##### 2.4.2.4 Protective effects of polysaccharides against H_2_O_2_^−^induced injury

According to the method of Wang et al. (18), after culturing RAW264.7 cells for 4 h, the medium was removed. Then, different concentrations of polysaccharide solutions were added into the wells to make their final concentrations were 0, 12.5, 25, and 50 μg/mL, respectively. The RAW264.7 cells were incubated for 24 h. The supernatant was removed after centrifugation (25°C, 4,000 rpm/min, 10 min), and H_2_O_2_ solution (final concentration 1 mmol/mL) was added. The cells were incubated for another 2 h. Normal cells cultured in medium with no additions were selected as control. H_2_O_2_ treatment group were set up by the addition of H_2_O_2_ solution (final concentration 1 mmol/mL). Finally, 30 μL of the MTT stock solution (5 mg/mL) was added, and the other steps followed were the same as those given in 2.4.2.2.

### 2.5 Structural characterization of GR polysaccharides

#### 2.5.1 Relative molecular weight and monosaccharide composition analysis

The relative molecular weight of polysaccharides was determined by high-performance gel permeation chromatography (HPGPC) using methods such as Meng et al. (19). The chromatographic conditions adopted were as follows: column: BRT105 − 104-102 (300 mm × 8 mm, 5 μm) tandem gel column; mobile phase: 0.05 mol/L NaCl; volumetric flow rate: 0.6 mL/min; column temperature: 40°C; injection volume: 20 μL. Aqueous stock solutions (5 mg/mL) of dextran with known molecular weights (5,000, 11,600, 23,800, 48,600, 80,900, 148,000, 273,000, 409,800, 667,800 Da) were injected into HPGPC for the construction of molecular weight-retention time calibration curve by plotting the logarithm of the molecular weight versus the retention time of each analyte. The polysaccharides were accurately weighed and dissolved to prepare a 5 mg/mL solution. This solution was then centrifuged at 12,000 rpm for 10 min, and the resulting supernatant was filtered through a 0.22 μm microporous membrane. The filtered sample was subsequently transferred to a 1.8 mL injection vial for analysis under the same conditions. Using the standard curve, a calculation formula was derived to determine the molecular weight of each sample.

The polysaccharide sample fraction was prepared by referring to the method of Qiao et al. (20). The polysaccharide (5.0 mg) was weighed precisely, added to 2 mL of trifluoroacetic acid (TFA, 4 mol/L), stirred at 120°C for 6 h until the solution became clear and transparent, and cooled to room temperature. The residual TFA in the sample was dried in a vacuum drying oven then was dissolved with 1.0 mL water. After centrifugation (13,000 rpm, 5 min), the supernatant was collected for PMP derivatization. PMP derivatization of monosaccharides was carried out as described previously with proper modification (21, 22). Briefly, 100 μL acid hydrolysate was mixed with 200 μL 0.5 M PMP methanolic solution and 100 μL ammonia water. This mixture was placed in a 70°C water bath for 30 min and cooled. Subsequently, 100 μL glacial acetic acid were added to neutralize the reaction solvent and 500 μL chloroform were successively added to remove the residual PMP reagents. The supernatant liquid was retained after vigorous shaking. The extraction procedure was repeated in triplicate. The upper aqueous phase was collected, filtered (0.45 μm), and set aside for use. The standard solutions, including Man, Rha, GlcA, GalA, Glc, Gal, Xyl, and Ara (23), were also treated as described above, subsequently determined through high-performance liquid chromatography.

The derivatized monosaccharide and polysaccharide samples were detected using a C18 column (4.6 × 250 mm, 5 μm) with a Shimadzu LC-20AB system at 245 nm. The mobile phase comprised 0.1 mol/L phosphate buffer (pH 6.7) and acetonitrile at a 1 mL/min flow rate. Finally, the equation for the monosaccharide standard curve (Supplementary Table S1) was established based on the peak area and molar mass of the monosaccharides. Using absolute quantitative methods, we determined the masses of different monosaccharides and calculated their molar ratios based on their respective molar masses.

#### 2.5.2 Purity, functional group, and triple helix structure identification

Ultraviolet (UV) (UV-2600, Shimadzu Corporation, Japan) spectra were scanned from 800 to 200 nm to identify nucleic acids and proteins in the polysaccharide samples.

Each polysaccharide was ground with dried KBr powder and pressed into wafers for infrared testing. According to the method of Luo et al. (24), An FT-IR spectrometer (Bruker TENSOR 27, Germany) scanning the wavelength range of 4,000-400 cm^−1^ was used to perform infrared testing.

The triple helix structure of the polysaccharides was measured using the Congo red method (25). Each polysaccharide solution was mixed with the Congo red solution, and the NaOH solution was then gradually added to the mixture to attain a final concentration of 0–0.5 mol/L. Using UV-2600, the maximum absorption wavelength (λ_max_) was determined in the 600–400 nm range.

#### 2.5.3 Ultrastructural observations

A trace amount of the sample powder was glued onto the conductive adhesive, and gold was sprayed on this powder at a 10-mA current for 45 s. Subsequently, the images were captured using a scanning electron microscope [TESCAN (China) Co., Ltd., TESCAN MIRA LMS] at 1,000× magnification. The sample images were observed at an accelerating voltage of 3 kV.

#### 2.5.4 Analysis of glycosidic bond types

The method of Needs and Selvendran (26) was used for the methylation analysis of polysaccharides to determine the type of glycosidic bond present in the polysaccharides. Dried samples (10 mg) underwent methylation reactions according to the kit (Borealis Biotechnology Co., Ltd.) instructions. The methylated polysaccharides were extracted with dichloromethane and hydrolyzed with TFA. The hydrolyzed products were reduced with NaBH_4_ (2 mol/L, 500 μL). Acetylation with acetic anhydride (2.5 mL) produced partially methylated glycol acetates, which were analyzed using a GC–MS (Agilent 7890B-5977A) equipped with DM-201MS (30 m × 0.25 mm × 0.25 μm). The program established was as follows: the initial column temperature was 100°C for 2 min, which was then increased to 260°C at 3°C /min and maintained for 5 min. The transfer line temperature was maintained at 260°C. An electron bombardment ion source was used. The electron energy was 70 eV, and the ion source temperature, detector temperature, and quadrupole temperature were 230, 106, and 150°C, respectively; and the mass scanning range was 30–600 m/z. In total, 2.14 scans were performed in every 1 s.

#### 2.5.5 Nuclear magnetic resonance spectral analysis

The polysaccharide was completely dissolved in 0.5 mL of heavy water to prepare a supersaturated solution. The supernatant was centrifuged (25°C, 10,000 rpm/min, 5 min) and placed in a nuclear magnetic resonance (NMR) tube. The one-dimensional NMR spectra (^1^H NMR, ^13^C NMR) and two-dimensional spectra (^1^H-^1^H COSY, HSQC, HMBC, and NOESY) were determined using the 600 MHz NMR instrument (Bruker, United States, model: Bruker Avance III 600) according to the method described by Zhu et al. (27, 28).

### 2.6 Statistical analysis

All of the data from triplicate determinations were expressed as means ± standard deviation. SPSS 17.0 software was used for the analysis of variance (ANOVA) by Duncan’s multiple-range test. Design-Expert 8.0.6 software was used for RSM and ANOVA. GraphPad Prism 8 software was used for plotting.

## 3 Results and discussion

### 3.1 Response surface analysis and validation

#### 3.1.1 Model fitting and optimization of crude polysaccharide extraction

As GR polysaccharides possess antidepressant, antioxidant, hypolipidemic, anticancer, and other properties, maximizing their extraction is a crucial prerequisite for developing GR polysaccharide products. Most polysaccharides in nature have higher solubility and stability in hot water, and the hot water extraction method minimizes damage to the polysaccharides, avoiding excessive chemical modification of the samples (29). Starting with hot water for the initial extraction, this would possibly denature some proteins and remove them by centrifugation (30). In addition, hot water extraction to obtain polysaccharides seemed to be more beneficial option with stronger bioactivity (31). However, the hot water extraction method has notable drawbacks, including being time-consuming and exhibiting a low extraction efficiency. Consequently, optimizing this method to enhance the extraction rate of polysaccharides is crucial for both this study and practical production applications.

According to the single-factor experimental results, the crude polysaccharide extraction rate was higher at an extraction temperature of 60°C, an extraction time of 30 min, and a liquid-to-material ratio of 20 mL/g (Supplementary Figure S2). In a one-way study of extraction time, the crude polysaccharide extraction rate increased and then decreased as the extraction time was extended, which may be because heating conditions can increase polysaccharide solubilization in a short period (46). By contrast, as the heating time was extended, polysaccharides were degraded, which led to a decrease in the polysaccharide yield (32). The results of the BBD experimental design and RSM experiments were as follows (Table 2). The experimental data were analyzed through multiple regression, and the dependent variable can be presented using the following second-order polynomial equation: Y_SGCPs_ = −227.225 + 7.247A + 1.11033B + 0.76425C + 8.33333 × 10^−4^AB − 0.00165 AC − 0.0021 BC − 0.0592A^2^ − 0.018578B^2^ − 0.019300C^2^; Y_NGCPs_ = −160.418 + 5.8038A + 1.0259B + 0.7616C − 0.0018333AB + 0.009125 AC + 0.00598333 BC − 0.051677A^2^ − 0.013257B^2^ − 0.034478C^2^.

#### 3.1.2 Analysis of response surface and validation of the model

The appropriateness of the models was determined through ANOVA, and the results were presented in Table 3. The *p*-values of 0.0007 and 0.0003 in the model for SGCPs and NGCPs, respectively, were low, which indicated that the model was significant. The correlation coefficient values (R^2^) were 0.9543 and 0.9653, respectively, and the adjusted coefficient of determination values (*R^2^*_Adj_) were 0.8956 and 0.9207, respectively, which further verified that the models were significant. Additionally, the coefficients of variation (C.V%) were 12.69 and 5.97%, respectively, which were both low and indicated that the experimental values were accurate and reliable. The aforementioned results indicate that the model was successfully established and is suitable for predicting optimal crude polysaccharide extraction.

**Table 3:** ANOVA of the regression model for the extraction rate of GR polysaccharides.

Sample	Source of variance	Equation of squares	Degrees of freedom	Mean square	*F*-value	*p*-value	Significance
SGCPs	Model	297.25	9	33.03	16.26	0.0007	***
	A	14.58	1	14.58	7.18	0.0316	*
	B	0.024	1	0.024	0.012	0.9162	
	C	23.05	1	23.05	11.35	0.0119	*
	AB	0.063	1	0.063	0.031	0.8657	
	AC	0.11	1	0.11	0.054	0.8235	
	BC	0.40	1	0.40	0.20	0.6718	
	A^2^	147.56	1	147.56	72.63	< 0.0001	***
	B^2^	73.57	1	73.57	36.21	0.0005	***
	C^2^	15.68	1	15.68	7.72	0.00274	**
	Residual	14.22	7	2.03			
	Lost proposal	7.77	3	2.59	1.61	0.3214	
	Pure error	6.45	4	1.61			
	Total deviation	311.47	16				
	Standard deviation	1.43		R^2^	0.9543		
	Average value	11.23		*R^2^*_Adj_	0.8956		
	C.V%	12.69		*R^2^*_Pred_	0.5683		
	PRESS	134.40		AP	10.538		
NGCPs	Model	331.14	9	36.79	21.64	0.0003	***
	A	50.20	1	50.20	29.53	0.0010	**
	B	2.86	1	2.86	1.68	0.2360	
	C	49.80	1	49.80	29.29	0.0010	**
	AB	0.13	1	0.13	0.074	0.7933	
	AC	3.33	1	3.33	1.96	0.2043	
	BC	3.22	1	3.22	1.90	0.2110	
	A^2^	112.44	1	112.44	66.14	< 0.0001	***
	B^2^	37.46	1	37.46	22.04	0.0022	**
	C^2^	50.05	1	50.05	29.44	0.0010	**
	Residual	11.90	7	1.70			
	Lost proposal	8.38	3	2.79	3.17	0.1471	
	Pure error	3.52	4	0.88			
	Total deviation	343.03	16				
	Standard deviation	1.3		R^2^	0.9653		
	Average value	21.83		*R^2^*_Adj_	0.9207		
	C.V%	5.97		*R^2^*_Pred_	0.5932		
	PRESS	139.54		AP	12.703		

**Figure 1:**
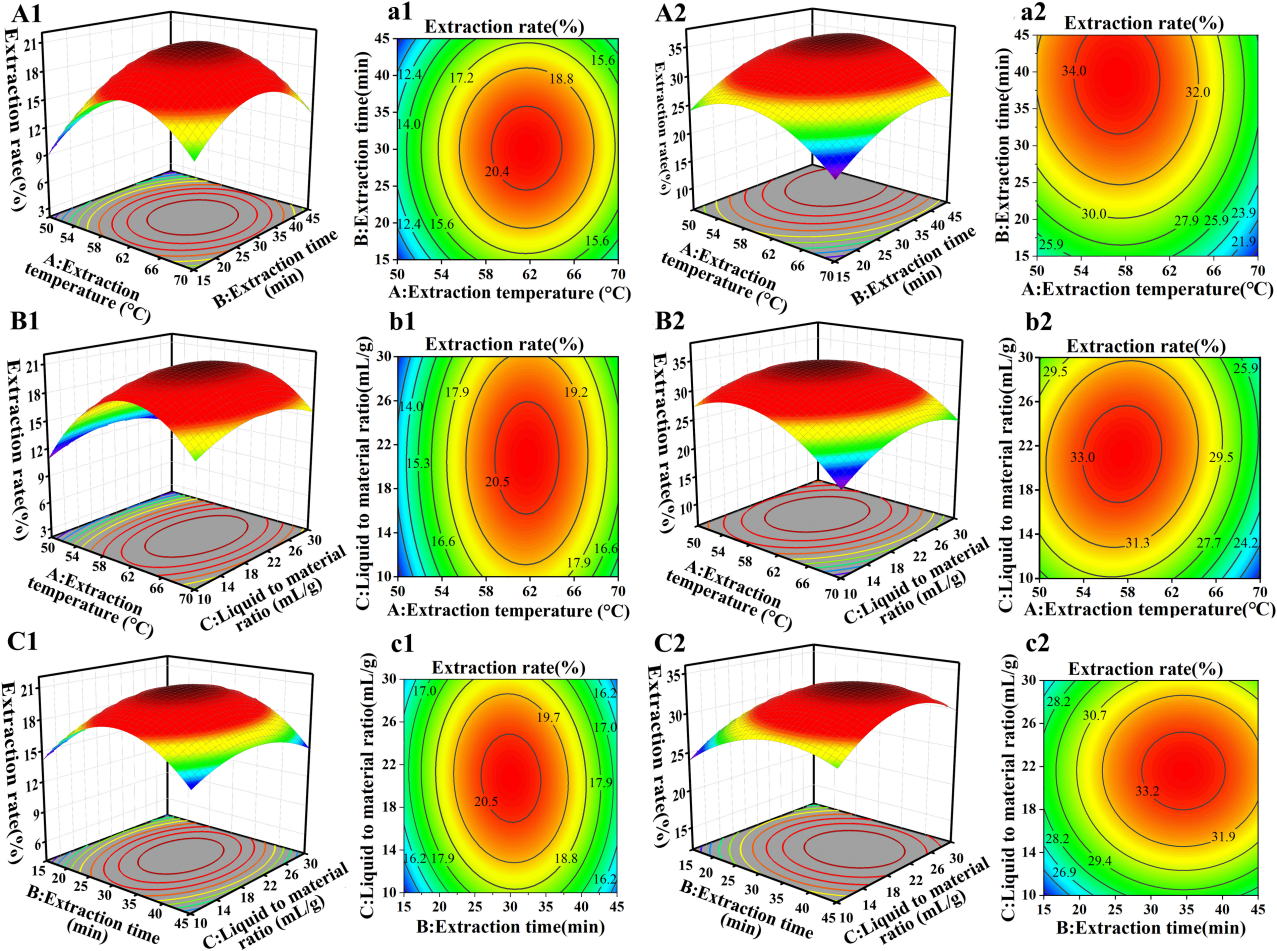
Response surface **(A1–C1)** and contour plots **(a1–c1)** of the extraction rate of sulfur-fumigated GR crude polysaccharides. Response surface plots **(A2–C2)** and contour plots **(a2–c2)** of the extraction rate of nonsulfur-fumigated GR polysaccharides.

Response surface plots and contour plots unveiled the effects of the two-factor interaction on the crude polysaccharide extraction rate. Polysaccharide extraction from GR by using RSM has been reported more often, but different researchers have obtained different results. For example, Chen et al. (33) found that the optimal conditions for extracting polysaccharides from GR were an extraction temperature of 74°C, an extraction time of 66 min, and a liquid-to-material ratio of 54 mL/g, the extraction rate was 6.11% ± 0.13%. In this study, the optimal extraction conditions for SGCPs (NGCPs) were an extraction temperature of 67°C (64°C), an extraction time of 31 min (32 min), and a liquid-to-material ratio of 15 mL/g (17 mL/g) (Figure 1).

The predicted and actual extraction yields for SGCPs were 15.48 ± 1.08% and 15.04 ± 1.91%, respectively, while the predicted and actual yields for NGCPs were 25.54 ± 1.54% and 25.67 ± 1.27%, respectively (Supplementary Figure S3). The results showed a high degree of agreement between the measured values and the predicted values, further confirming that the model has high stability and feasibility. This indicates that the optimized extraction process parameters in this study are accurate and reliable. The actual extraction rate of SGCPs was significantly reduced by 10.63% compared with that of NGCPs (Supplementary Figure S3). This was because of starch degradation caused by acidic conditions resulting from sulfur fumigation (12), which led to lower polysaccharide extraction. Sulfur fumigation reduces the extraction efficiency and rate of GR polysaccharides.

### 3.2 Isolation and purification of GR polysaccharides

**Figure 2:**
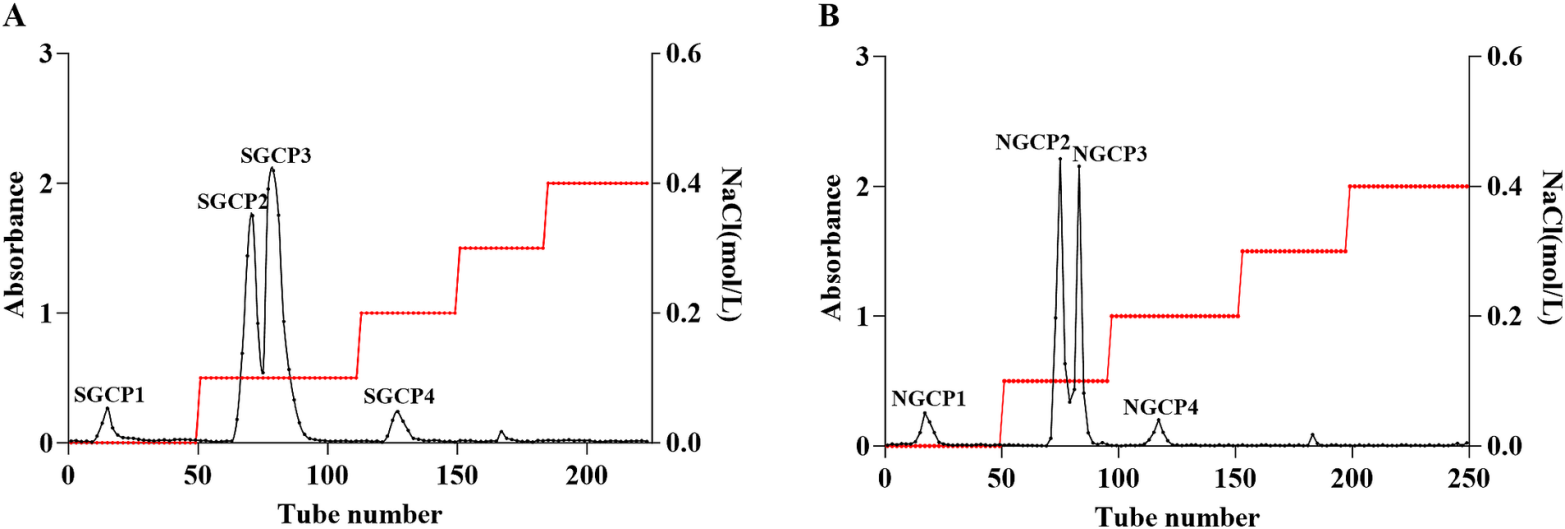
DEAE-52 cellulose column chromatography elution profiles. **(A)** Sulfur-fumigated GR crude polysaccharides. **(B)** Nonsulfur-fumigated GR crude polysaccharides.

The elution curves displayed that both SGCPs and NGCPs can be separated into four polysaccharide components. SGCP1, SGCP2, SGCP3, and SGCP4 accounted for 4.9, 40.2, 49.4, and 5.5%, respectively. NGCP1, NGCP2, NGCP3, and NGCP4 accounted for 7.4, 45.8, 41.2, and 5.6%, respectively. In this study, SGCP2 and SGCP3 and NGCP2 and NGCP3, which exhibited a higher proportion, were collected. These four fractions were then purified through dialysis, lyophilization, and Sephadex G-200 gel chromatography (Figure 2).

The total sugar content of three batches of purified polysaccharides was determined using the phenol-sulfuric acid method. The results showed that the total sugar contents of SGCP2 and SGCP3 were 94.2 and 89.5%, respectively; the total sugar contents of NGCP2 and NGCP3 were 81.0 and 85.1%, respectively (Table 4). This indicates that the polysaccharide components became more concentrated after purification. Furthermore, sulfur fumigation reduced the total sugar content of GR polysaccharides, which is consistent with the results of Wu et al. (34). This phenomenon can be attributed to the hydrolysis of starch side chains into sugar substances due to sulfur fumigation (12).

**Table 4:** Total sugar content and molecular weight of sulfur-fumigated and nonsulfur-fumigated GR polysaccharides.

Sample	Total sugar content (%)	RT (min)	lgMw	Mw (Da)
SGCP2	94.2 ± 0.7 a	39.048	4.5	31,027 ± 1791 c
SGCP3	89.5 ± 1.8 b	32.025	5.9	727,650 ± 3,928 a
NGCP2	81.0 ± 2.9 d	38.493	4.6	39,812 ± 1,365 b
NGCP3	85.1 ± 0.4 c	38.483	4.6	39,991 ± 2013 b

Different lowercase letters indicate significant differences. *p* < 0.05. RT, retention time; Mw, molecular weight.

### 3.3 Effect of sulfur fumigation on the antioxidant activity of GR polysaccharides

#### 3.3.1 Effect of sulfur fumigation on the *in vitro* antioxidant activity of GR polysaccharides

**Figure 3:**
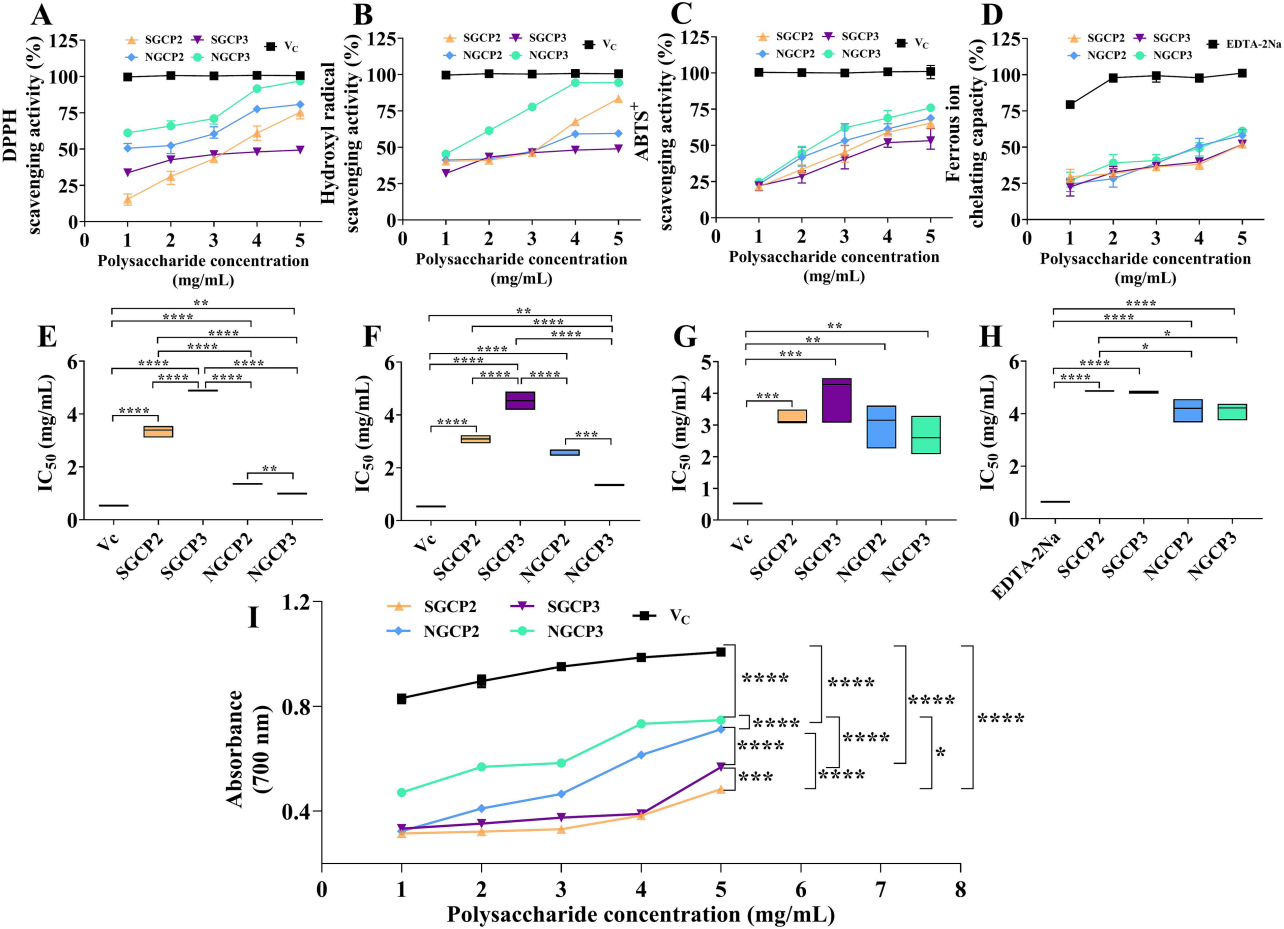
*In vitro* scavenging capacity of different GR polysaccharides for scavenging reactive oxygen radicals. **(A)** DPPH radical-scavenging activity, **(B)** hydroxyl radical-scavenging activity, **(C)** ABTS^+^ radical-scavenging activity, **(D)** ferrous ion-chelating capacity, **(E)** IC_50_ value for DPPH radical-scavenging activity, **(F)** IC_50_ value for hydroxyl radical-scavenging activity, **(G)** IC_50_ value for ABTS^+^ radical-scavenging activity, **(H)** Fe^2+^ chelation capacity of the IC_50_ values, and **(I)** total reducing power. The respective values are expressed as the mean ± SD (*n* = 3). *, **, ***, **** indicate significant differences at *p* < 0.05, *p* < 0.01, p < 0.001, *p* < 0.0001 levels, respectively.

Antioxidant activity is the most researched model of polysaccharides. Therefore, the present study used the antioxidant activity model to comparatively investigate the effect of sulfur fumigation on GR polysaccharide activity. Results indicated that the DPPH, hydroxyl, and ABTS^+^ radical scavenging capacities of NGCP2, NGCP3, SGCP2, and SGCP3 increased with polysaccharide concentration. Undoubtedly, the scavenging activity of Vitamin C was higher than the NGCPs and SGCPs. Notably, NGCP3 (NGCP2) at 5 mg/mL achieved a DPPH scavenging rate of 96.89% (80.76%) and an IC_50_ of 0.994 (1.357) mg/mL. The NGCPs were better than the SGCP3 (SGCP2), which were 49.31% (75.38%) at the same concentration, and the IC_50_ was 4.887 (3.348) mg/mL (Figures 3A,E).

As with the above results, although the NGCPs and SGCPs’s scavenging of hydroxyl radicals increased with the increase in their concentration, the fact that the IC_50_ value of the NGCP3 and NGCP2 (1.346 and 2.602 mg/mL) were lower than that of the SGCP3 and SGCP2 (3.536 and 3.082 mg/mL) suggested that the scavenging effect of the NGCPs was superior to that of the SGCPs (Figures 3B,F). The ability of SGCPs to scavenge ABTS^+^ radicals was found to be 1.63 to 22.32% lower than that of NGCPs at concentrations ranging from 1 to 5 mg/mL. Furthermore, the IC_50_ values for SGCP3 and SGCP2 were 3.946 mg/mL and 3.216 mg/mL, respectively, which are higher than the IC_50_ values for NGCP3 and NGCP2, which were 2.656 mg/mL and 3.006 mg/mL, respectively (Figures 3C,G).

The Fe^2+^-chelating abilities of NGCPs, and SGCPs improved with an increase in the polysaccharide concentration. Their IC_50_ values were as follows: NGCP2 (3.006 mg/mL), NGCP3 (4.114 mg/mL), SGCP3 (3.216 mg/mL), SGCP2 (4.863 mg/mL), while the IC_50_ value of EDTA-2Na was 0.645 mg/mL (Figures 3D,H). The total reducing capacities of the four fractions at the same concentration were in the following order: NGCP3 > NGCP2 > SGCP3 > SGCP2. Compared with NGCP, SGCP decreased the total reducing capacity from 0.142 to 0.263 (Figure 3I).

Compared with GR polysaccharides in Hanzhong, Shaanxi, both SGCP and NGCP in this study exhibited a higher ability to scavenge reactive oxygen species (ROS) at the same treatment concentration (30). Based on the results of this study, GR polysaccharides displayed certain antioxidant potential regardless of whether they were subjected to sulfur fumigation, but sulfur fumigation reduced the antioxidant activity of GR polysaccharides. The antioxidant activity of polysaccharides is mainly related to their chemical composition, molecular weight, and structural characteristics (35). The different monosaccharide compositions allow polysaccharides to react with free radicals in various ways, thereby enhancing their ability to scavenge free radicals. Therefore, elucidating the impact of sulfur fumigation on the structure of polysaccharides is key to understanding the increase in molecular weight and the decrease in antioxidant activity.

#### 3.3.2 Effect of sulfur fumigation on the antioxidant capacity of GR polysaccharides at the cellular level

**Figure 4:**
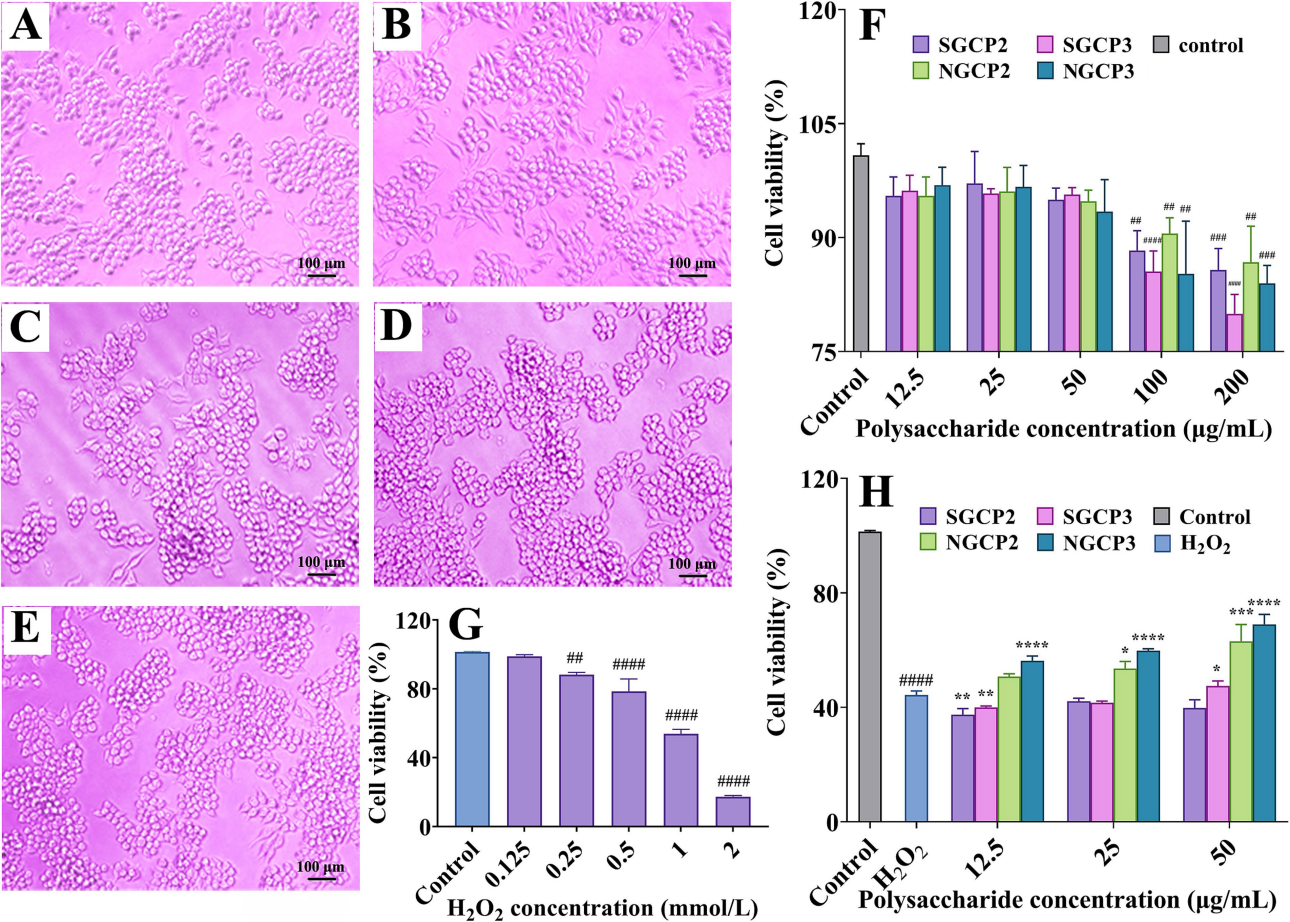
**(A–E)** The effect of polysaccharides on cell proliferation (100x). **(A)** SGCP2, **(B)** SGCP3, **(C)** NGCP2, **(D)** NGCP3, and **(E)** Control (polysaccharide concentration of 50 μg/mL). After 24 h of cell culture, four types of polysaccharides were added separately, and the cell state was further cultured for 24 h. **(F)** Effect of four fractions of polysaccharides on RAW264.7 cell viability. **(G)** Effect of H_2_O_2_ on RAW264.7 cell viability. **(H)** Effect of four fractions of polysaccharides on H_2_O_2_-induced RAW264.7 cell viability damage. Results are expressed as the mean ± SD (*n* = 3). ##*p* < 0.01 vs. Control, ####*p* < 0.0001 vs. Control; *p < 0.05 vs. H_2_O_2_ group alone, **p < 0.01 vs. H_2_O_2_ group alone, ***p < 0.001 vs. H_2_O_2_ group alone, ****p < 0.0001 vs. H_2_O_2_ group alone.

The four polysaccharide fractions (SGCP2, SGCP3, NGCP2, and NGCP3) did not significantly affect RAW264.7 cell viability at 12.5, 25, and 50 μg/mL, whereas 100 and 200 μg/mL treatments inhibited cell viability. Therefore, 12.5, 25, and 50 μg/mL were selected as the treatment concentrations (Figure 4F). Cell viability decreased as the exogenous H_2_O_2_ concentration increased. The survival rates after treatment with 0.125, 1, and 2 mmol/L H_2_O_2_ were 98.82, 53.85, and 17.28%, respectively (Figure 4G). Low H_2_O_2_ concentrations caused no significant damage to the cells, whereas high H_2_O_2_ concentrations caused cell death. Therefore, 1 mmol/L H_2_O_2_ was selected to treat the cells and investigate the effect of polysaccharides on H_2_O_2_-induced RAW264.7 cell damage.

NGCP or SGCP had no significant effect on cell morphology after 24 h of administration (Figures 4A–E). The RAW264.7 cell survival rate under H_2_O_2_ treatment was 44.30%. NGCP2 and NGCP3 protected the RAW264.7 cells against H_2_O_2_-induced oxidative damage, which was manifested through cell viability enhancement with an increase in the treatment concentration (Figure 4H). At 50 μg/mL, cell viabilities under NGCP2 and NGCP3 treatments were 63.12 and 70.00%, respectively, which were 18.82 and 24.70% higher than those under H_2_O_2_ treatments, respectively (Figure 4H). These results were similar to those of Huang et al. (36), which indicated the cytoprotective potential of GR polysaccharides against H_2_O_2_-treated cellular damage. Under H_2_O_2_ treatments, cell viability enhancement by SGCP2 and SGCP3 was significantly lower than that by NGCP2 and NGCP3 (Figure 4H). Sulfur fumigation thus reduced the protective effect of GR polysaccharides against H_2_O_2_-induced RAW264.7 cell damage. This once again confirms that sulfur fumigation can reduce the antioxidant activity of GR polysaccharides. This study is the first to identify that sulfur fumigation can reduce the protective effect of GR polysaccharides against oxidative stress-induced cell damage.

### 3.4 Analysis of the relative molecular weight and monosaccharide composition of GR polysaccharides

The antioxidant activities of polysaccharides are influenced by the molecular weight. Low-molecular-weight polysaccharides exhibit higher antioxidant activities (37, 38). In the study, all chromatograms of the purified polysaccharide fractions obtained through HPGPC exhibited a single symmetric elution peak (Supplementary Figure S4), indicating a relatively uniform distribution of molecular weights. The regression equation of lgMw versus retention time (t) was: lgMw = −0.1951 t + 12.11 (R^2^ = 0.996). Based on the results of Supplementary Figure S4, the molecular weight of each sample was calculated by the regression equation. The results showed that the molecular weight (Mw) of SGCP3 and NGCP3 were 727,650 and 39,991, respectively (Table 4). This mutually confirms the results of its antioxidant activity, that is, the molecular weight of polysaccharides is too large to expose antioxidant active components (39).

**Figure 5:**
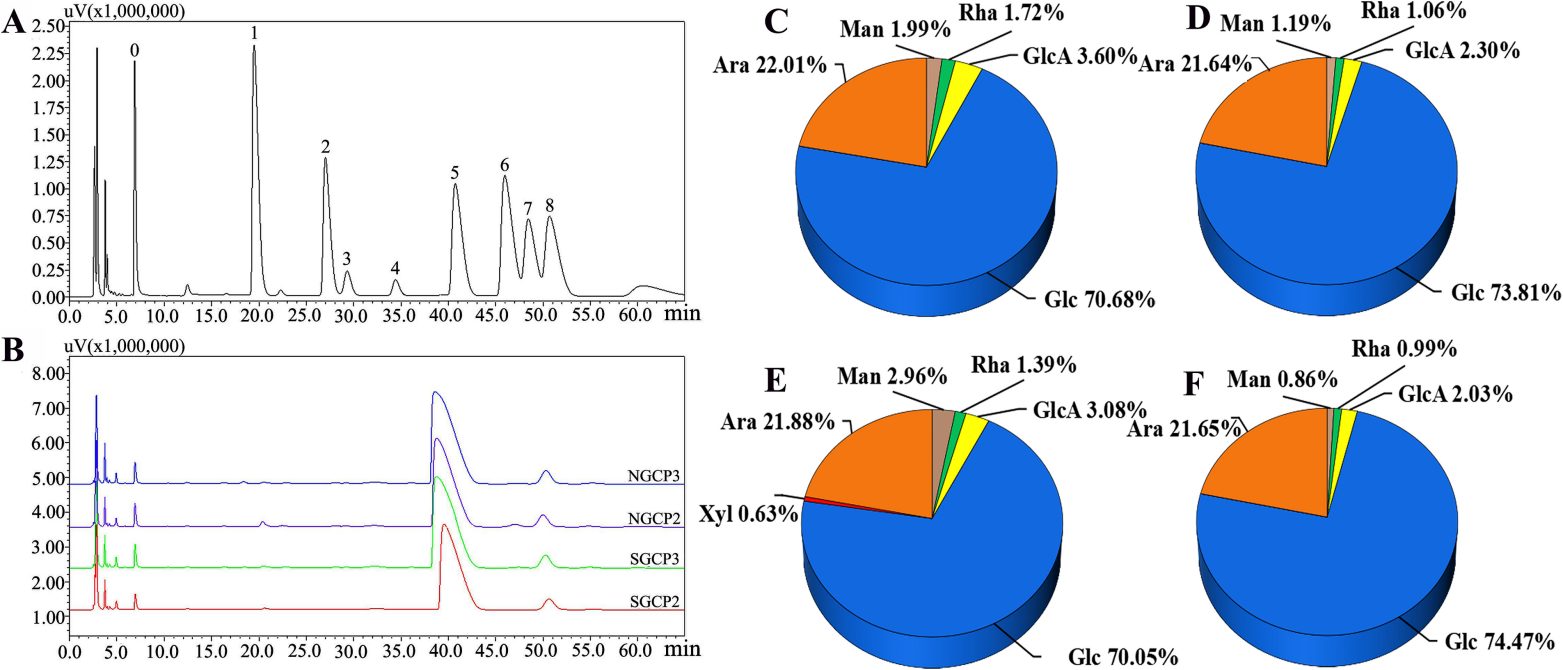
Monosaccharide composition of sulfur-fumigated and nonsulfur-fumigated GR polysaccharides. **(A)** HPLC plots of monosaccharide standard derivatives (0. PMP, 1, Man, 2, Rha, 3, GlcA, 4, GalA; 5, Glc; 6, Gal; 7, Xyl; 8, Ara). **(B)** HPLC plots of derivatives of sulfur-fumigated and nonsulfur-fumigated GR polysaccharides. **(C–F)** Shows the monosaccharide composition and their molar ratios of SGCP2, SGCP3, NGCP2, and NGCP3, respectively.

Previous studies have shown that different components of polysaccharides possess varying biological activities (40). According to Zhang et al. (41), the higher the glucose content in the monosaccharide composition of polysaccharides, the higher the polysaccharide antioxidant activity. Figure 5 present the results of the monosaccharide composition and molar ratio of each polysaccharide. SGCP2, SGCP3, NGCP2 and NGCP3 were primarily composed of Glc with molar percentages of 70.68, 73.81, 70.05, and 74.47%, respectively. Teng et al. (42) isolated polysaccharides from *Chenopodium quinoa*, primarily composed of glucose, which exhibited dose-dependent antioxidant activity. In SGCP2, the ratio of the ratio of Glc to Ara, GlcA, Man, and Rha is 41.09:12.8:2.09:1.16:1. In SGCP3, the corresponding ratio is 69.63:20.42:2.17:1.12:1. For NGCP2, the ratio is 50.4:15.74:2.22:2.13:1, while in NGCP3, it is 75.22:21.87:2.05:0.87:1. It can be seen that sulfur fumigation significantly reduced the proportion of Glc and Ara in the polysaccharide components. Therefore, the differences of molecular weight and monosaccharide composition may be part of the reason why the NGCPs showed more antioxidant activity than the SGCPs.

### 3.5 Identification of polysaccharide purity, functional groups, and triple helix structure

**Figure 6:**
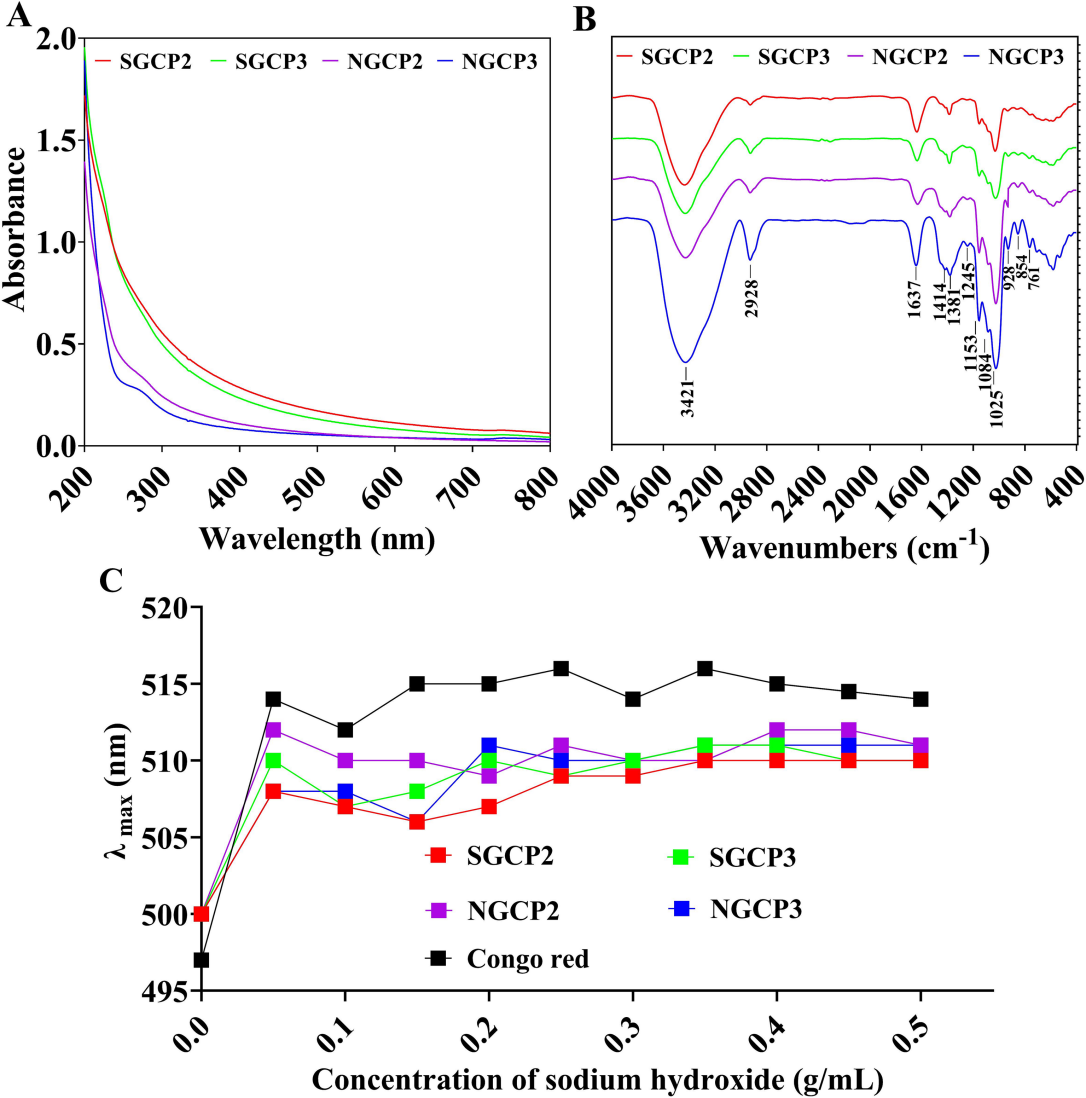
**(A)** UV spectra. **(B)** Fourier infrared spectra. **(C)** Congo red experiments of sulfur-fumigated and nonsulfur-fumigated GR polysaccharides.

The four component polysaccharides (SGCP2, SGCP3, NGCP2, and NGCP3) exhibited no significant absorption peak near 260 nm and 280 nm, which indicated that they contained no proteins or nucleic acids (Figure 6A).

According to the FT-IR spectra of the four polysaccharides (SGCP2, SGCP3, NGCP2, and NGCP3), the absorption peak at 3,421 cm^−1^ was attributable to the O-H stretching vibration (Figure 6B). The absorption peak at 2,928 cm^−1^ was attributable to the asymmetric C-H stretching vibration, and the absorption peak near 1,637 cm^−1^ was attributable to the -C=O bond stretching vibration. The three characteristic absorption peaks at 1,153, 1,084, and 1,025 cm^−1^ indicated that the four polysaccharide fractions were of the pyran type. The absorption peak at 1,025 cm^−1^ is a characteristic absorption peak of glucose, which indicates that glucose is the main component monosaccharide of the polysaccharide. The absorption peak at 928 cm^−1^ is the characteristic absorption peak of C-O-C, which is a typical characteristic absorption peak of D-pyran type glucose. The absorption peak at 854 cm^−1^ indicates that the glycosidic bond type is mainly an *α*-configuration. The GR polysaccharides SGCP2, SGCP3, NGCP2, and NGCP3 are thus all α-D-glucopyranose.

The maximum uptake of Congo red did not redshift with an increase in the NaOH concentration from 0.05 to 0.25 mol/L. This indicated that none of the SGCP2, SGCP3, NGCP2, and NGCP3 existed in a triple helix conformation (Figure 6C).

### 3.6 Ultrastructural analysis of polysaccharides

**Figure 7:**
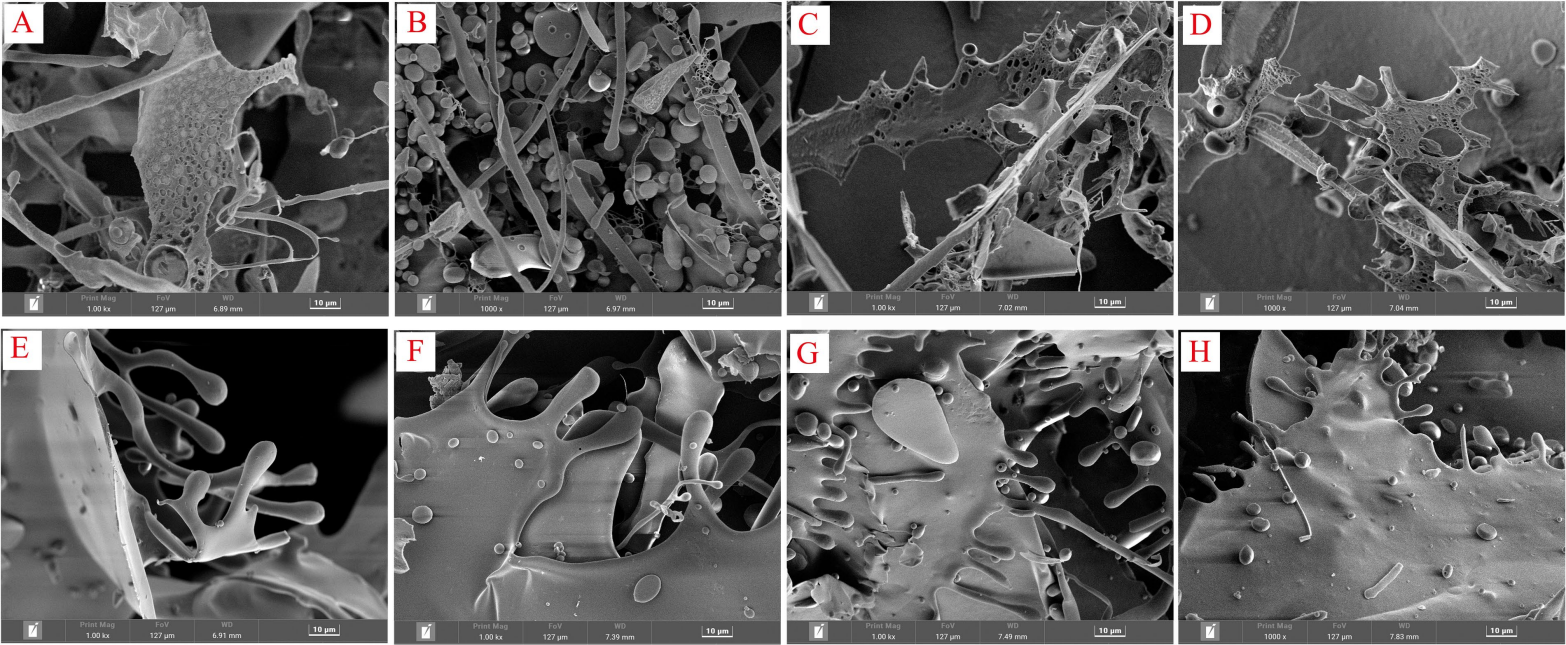
Scanning electron micrographs of sulfur-fumigated and nonsulfur-fumigated GR polysaccharides. **(A)** SGCP2-a, **(B)** SGCP2-b, **(C)** SGCP3-a, **(D)** SGCP3-b, **(E)** NGCP2-a, **(F)** NGCP2-b, **(G)** NGCP3-a, and **(H)** NGCP3-b (**A,B** are images of the same sample at different angles).

The biological activity of polysaccharides is often associated with their three-dimensional (3D) structure. Both SGCPs and NGCPs exhibited a lamellar structure. The surfaces of NGCPs were smoother, and the edges were connected by balls and rods (Figures 7E–H). However, the SGCPs exhibited more holes in the lamellar structure, which were present as a net (Figures 7A–D). Perhaps due to the sulfite formed after sulfur fumigation promoting hydrolysis, the structure of GR polysaccharides has changed (1). From the study of antioxidant activity, it can be found that the 3D structure of sulfurless smoked GR polysaccharides can significantly promote the improvement of polysaccharide activity (16).

### 3.7 Methylation analysis of polysaccharides

The mass spectra corresponding to the PMAA peaks of the four component polysaccharides (SGCP2, SGCP3, NGCP2 and NGCP3) were compared with those available on the standard spectral database. All four polysaccharides mainly contained three glycosidic bonds, namely D-Glc*p*-1→, →4)-D-Glc*p*-(1→, and →4,6)-D-Glc*p*-(1 → (Table 5; Supplementary Figures S5–S9). The molar percentage of each glycosidic bond calculated from the peak area of each monosaccharide showed that (1 → 4)-linked D-glucopyranosyl and (1 → 4,6)-linked D-glucopyranosyl were present in all four polysaccharides. The molar ratio of the glycan residue →4)-D-Glc*p-*(1 → was the largest of all the four polysaccharides, which suggested that it is the major linkage structure.

**Table 5:** Methylation analysis of polysaccharides SGCP2, SGCP3, NGCP2, and NGCP3.

Polysaccharide	Partially methylated glycol acetate	Sugar residue linkage	Mole fraction
SGCP2	2,3,4,6-Me_4_-Glc*p*	D-Glc*p*-1→	23.75%
	2,3,6-Me_3_-Glc*p*	→4)-D-Glc*p*-(1→	67.37%
	2,3-Me_2_-Glc*p*	→4,6)-D-Glc*p*-(1→	8.88%
SGCP3	2,3,4,6-Me_4_-Glc*p*	D-Glc*p*-1→	6.65%
	2,3,6-Me_3_-Glc*p*	→4)-D-Glc*p*-(1→	91.39%
	2,3-Me_2_-Glc*p*	→4,6)-D-Glc*p*-(1→	1.96%
NGCP2	2,3,4,6-Me_4_-Glc*p*	D-Glc*p*-1→	8.16%
	2,3,6-Me_3_-Glc*p*	→4)-D-Glc*p*-(1→	81.57%
	2,3-Me_2_-Glc*p*	→4,6)-D-Glc*p*-(1→	10.28%
NGCP3	2,3,4,6-Me_4_-Glc*p*	D-Glc*p*-1→	6.59%
	2,3,6-Me_3_-Glc*p*	→4)-D-Glc*p*-(1→	89.93%
	2,3-Me_2_-Glc*p*	→4,6)-D-Glc*p*-(1→	3.49%

### 3.8 Structural analysis of GR polysaccharides based on NMR spectroscopy

**Figure 8:**
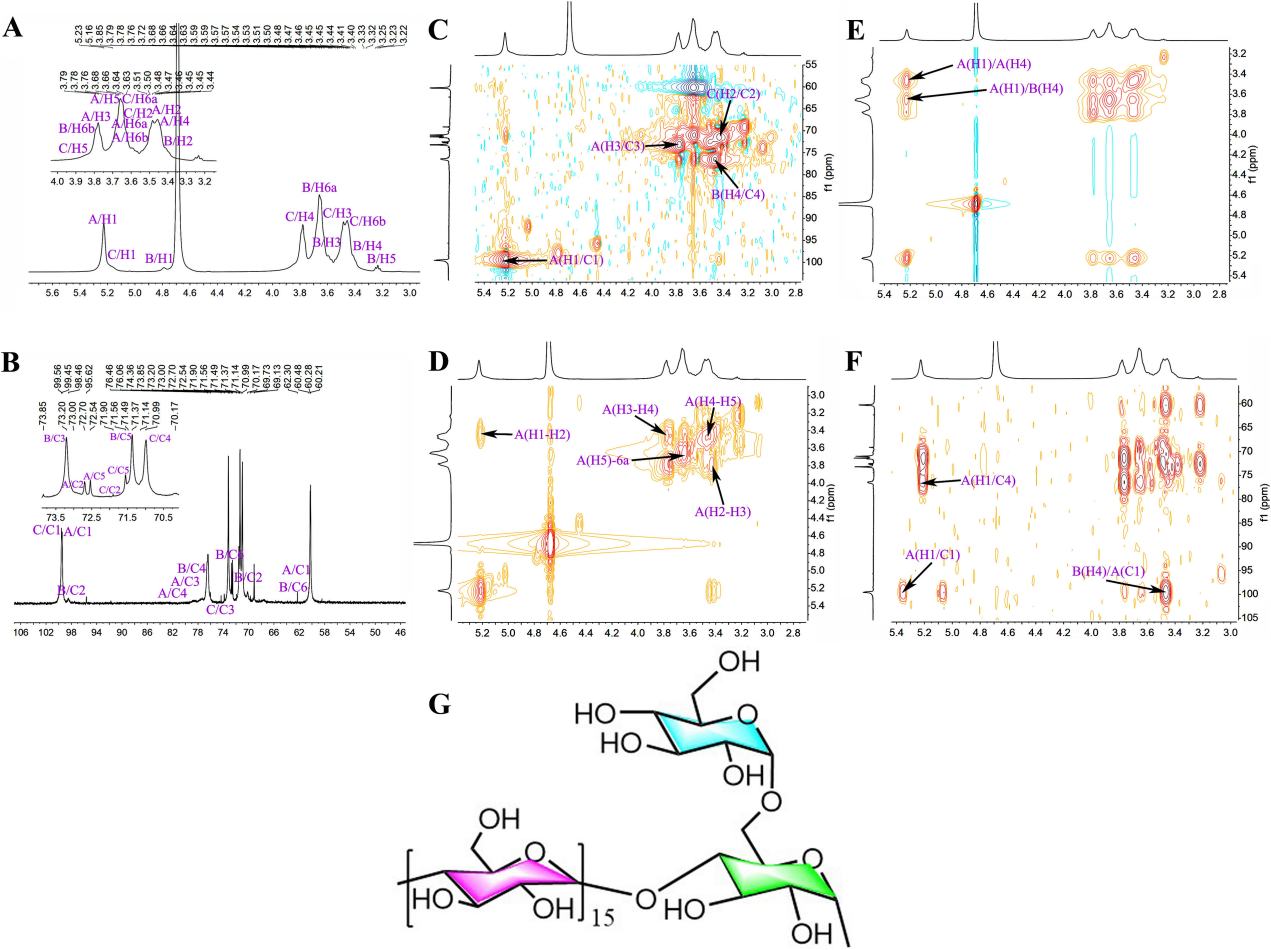
The NMR spectra and chemical structural formula of sulfur-fumigated GR polysaccharides SGCP3. **(A)** ^1^H, **(B)** ^13^C, **(C)** HSQC, **(D)** ^1^H-^1^H COSY, **(E)** NOESY, **(F)** HMBC, and **(G)** chemical structural formula.

SGCP3 and NGCP3 were selected for the NMR spectroscopy analysis because antioxidant activities between these two polysaccharides were significantly different in the antioxidant activity test. The ^1^H chemical shift of SGCP3 ranged from 3.00 to 5.50 ppm, and the H protons of sugar were in the range of *δ* 3.20–4.00 ppm. The major end-group proton peak signals were distributed in the 4.30–5.50 ppm region (Figure 8A). The ^1^C chemical shift ranged from 60.00 to 120.00 ppm, and the anomeric carbon region ranged from δ 93.00 to 105.00 ppm, and the major signal peaks were distributed in the 60.00–85.00 ppm region (Figure 8B). SGCP3 was mainly composed of glucose, which suggested that this polysaccharide was mainly a dextran. δ 101.03 in the HSQC was the anomeric carbon signal, and δ 5.22 was the corresponding anomeric hydrogen signal (Figure 8C). H1-2, H2-3, H3-4, H4-5, and H5-6a signals were 5.22/3.43, 3.43/3.78, 3.78/3.49, 3.49/3.64, and 3.64/3.69 in ^1^H-^1^H COSY, respectively, and the signals of H1, H2, H3, H4, H5, and H6 were δ 5.22, 3.43, 3.78, 3.49, 3.64, and 3.69, respectively (Figure 8D). The corresponding C1-6 signals were δ 100.57, 72.82, 74.19, 77.55, 72.44, and 61.29, respectively. Therefore, the signal was ascribed to the glycosidic bond →4)-*α*-Glc*p*-(1→. By combining NOESY (Figure 8E) and HMBC (Figure 8F), all glycosidic bond signals were assigned, and the results are shown in Supplementary Table S2. According to the HMBC profiles, the anomeric hydrogen of the glycosidic bond →4)-α-D-Glc*p*-(1 → exhibited a correlation signal peak with its C4, and the other anomeric carbon exhibited a correlation signal peak with its H4. →4)-α-D-Glc*p*-(1 → exhibited a correlation peak between the anomeric hydrogen of →4)-α-D-Glc*p*-(1 → and the C4 of the glycosidic bond →4,6)-α-D-Glc*p*-(1→, which suggested the presence of →4)-α-D-Glc*p*-(1 → 4)-α-D-Glc*p*-(1 → and →4)-α-D-Glc*p*-(1 → 4,6)-α-D-Glc*p*-(1 → linkages. The NOESY analysis revealed that the anomeric hydrogen of α- D-Glc*p*-(1 → exhibited a correlation signal peak with H6 of →4,6) -α- D-Glc*p*-(1→, which suggested the presence of α- D-Glc*p*-(1 → 4,6) -α-D-Glc*p*-(→ linkages. The anomeric hydrogen of →4)-α-D-Glc*p*-(1 → exhibited a correlation signal peak with its H4 and that the anomeric hydrogen of →4)-α-D-Glc*p*-(1 → displayed a correlation signal peak with H4. →4,6)-α-D-Glc*p*-(1 → showed a correlation signal peak with H4 of →4)-α-D-Glc*p*-(1 → 4)-α-D-Glc*p*-(1 → and →4)-α-D-Glc*p*-(1 → 4,6)-α-D-Glc*p*-(1 → linkages. On combining the results of the methylation analysis and hydrogen spectral integration, we found that the ratio of the sum of the quantities of (*α*-D-Glc*p*-1 → and →4)-α-D-Glc*p*-(1 to →4,6)-α-D-Glc*p*-(1 → was 16:1. Therefore, the main glycosidic bond structural linkage of the polysaccharide SGCP3 was inferred to be the main chain linkage of the glycosidic bond of →4)-*α*-D-Glc*p*-(1→, while the terminal group α-D-Glc*p*-(1 → was considered to be attached to the main chain through an O-6 bond. The deduced chemical structure of SGCP3 is presented in Figure 8G.

**Figure 9:**
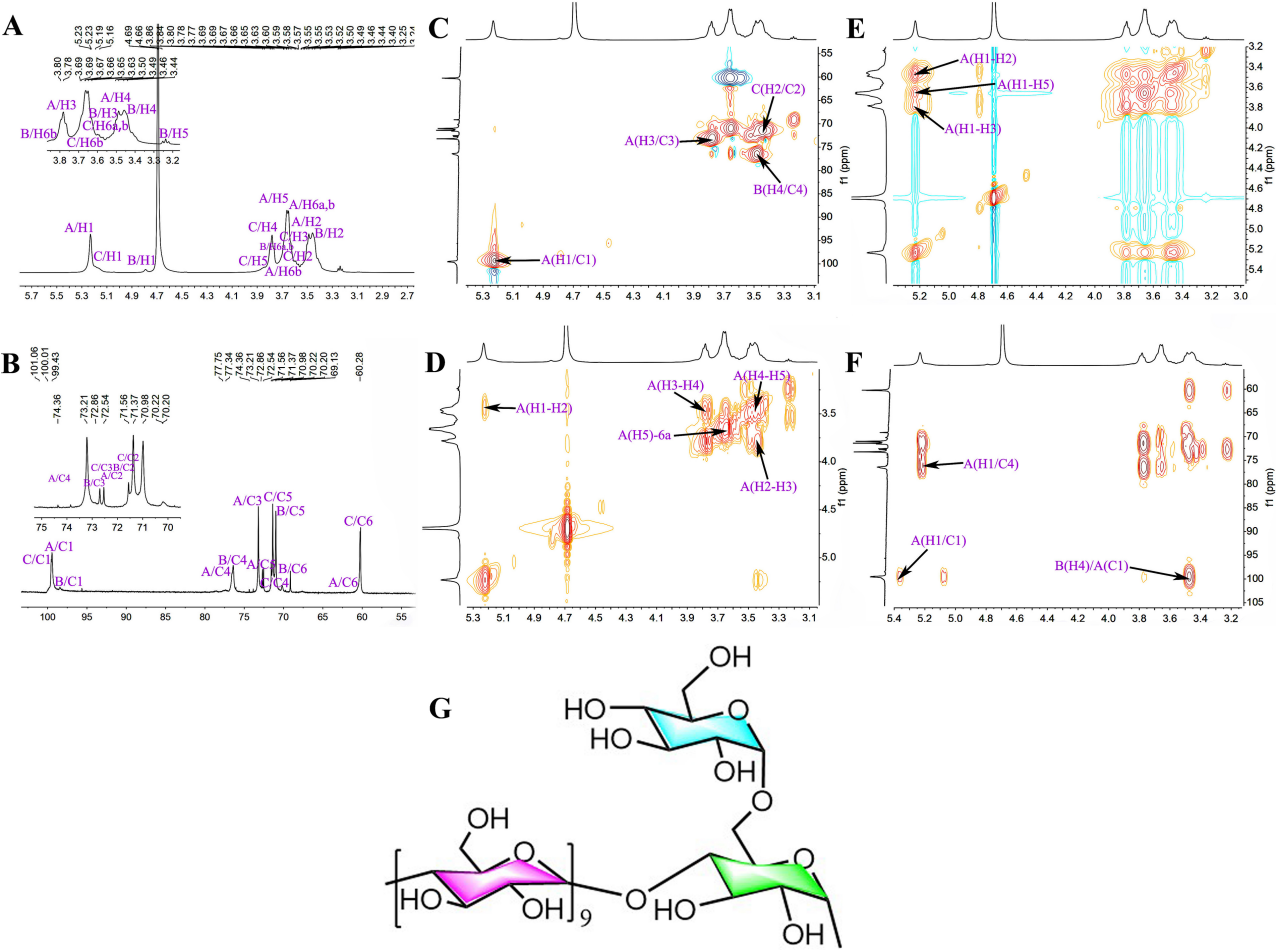
The NMR spectra and chemical structural formula of nonsulfur-fumigated GR polysaccharides NGCP3. **(A)** ^1^H, **(B)** ^13^C, **(C)** HSQC, **(D)** ^1^H-^1^H COSY, **(E)** NOESY, **(F)** HMBC, and **(G)** chemical structural formula.

The ^1^H chemical shifts of NGCP3 were mainly observed between 3.00 and 5.50 ppm, and the H protons of sugar ranged from *δ* 3.20 to 4.00 ppm, with the major end-substrate proton peaks distributed in the 4.30–5.50 ppm region (Figure 9A). The ^1^C chemical shifts were mainly centered in the range of 60.00–120.00 ppm. The anomeric carbons were distributed in the range of δ 93.00–105.00, with the major signal peaks observed in the 60.00–85.00 ppm range (Figure 9B). NGCP3 was mainly composed of glucose, which indicated that the polysaccharide was mainly a glucan. According to HSQC results, the inferred chemical shift of the anomeric carbon was δ 100.51, and the corresponding anomeric hydrogen signal was δ 5.23 (Figure 9C). The signals of H1-2, H2-3, and H3-4 in ^1^H-^1^H COSY were 5.23/3.47, 3.47/3.78, and 3.78/3.48, respectively (Figure 9D), and the signals of H1-4 were inferred to be δ 5.23, 3.47 3.78, and 3.48, respectively. δ 5.23 exhibited correlation peaks with 3.47, 3.70, and 3.78, and H5 was 3.70 ppm, as observed through NOESY (Figure 9E). The corresponding chemical shifts for C5 and C6 were 72.45 and 61.81, corresponding to δ 3.59 for H6a. Therefore, the signal was attributed to →4)-*α*-Glc*p*-(1→. By combining NOESY and HMBC (Figure 9F), all glycosidic bond signals were assigned, and the results are shown in Supplementary Table S2. The HMBC mapping analysis revealed that the anomeric hydrogen of the glycosidic bond →4)-*α*-D-Glc*p*-(1 → exhibited a correlation signal peak with its C4. The anomeric carbon demonstrated a correlation signal peak with its H4. The isocaprocarbons have correlated signal peaks with their own H4, as well as the →4)-*α*-D-Glc*p*-(1 → isocaprocarbons have correlated peaks with the →4,6)-α-D-Glc*p*-(1→’ C4, which indicated that the presence of →4)-*α*-D-Glc*p*-(1 → 4)-*α*-D-Glc*p*-(1 → and →4)-*α*-D-Glc*p*-(1 → 4,6)-*α*-D-Glc*p*-(1 → linkages. The NOESY analysis revealed that the anomeric hydrogen of *α*- D-Glc*p*-(1 → exhibited a correlation signal peak with H6 of →4,6) -*α*- D-Glc*p*-(1→, which suggested the presence of α- D-Glc*p*-(1 → 4,6) -α-D-Glc*p*-(→ linkages. The linkage mode of →4)-*α*-D-Glc*p*-(1 → with →4,6)-α-D-Glc*p*-(1 → of H4 had a correlation peak. This indicated the existence of a → 4)-α-D-Glc*p*-(1 → 4,6)-α-D-Glc*p*-(1 → linkage. On combining the results of the methylation analysis and hydrogen spectrum integration, we found that the ratio of the sum of the quantities of (α-D-Glc*p*-1 → and →4)-α-D-Glc*p*-(1 to →4,6)-α-D-Glc*p*-(1 → was 10:1. Therefore, the main glycosidic bonding structure of the polysaccharide was inferred to be linked in such a way that the glycosidic bond →4)-α-D-Glc*p*-(1 → was the main chain linkage, whereas the end-group α-D -Glc*p*-(1 → was attached to the main chain through an O-6 bond. Figure 9G presents the deduced chemical structure formula of NGCP3.

The type and connection mode of glycosidic bonds all affect antioxidant activities of polysaccharides. The glycosidic bond composition of Huo et al. (43) found GR polysaccharides (GEP4) and Guan et al. (44) found that (GEP1) both have α-D-Glc*p*-(1→, →4)-α-Glc*p*-(1, and →4)-6-α-Glc*p*-(1→. The molar ratio of→4)-α-Glc*p*-(1 was the highest (82.66%), followed by those of α-D-Glc*p* -(1 → (7.59%) and →4)-6-α- Glc*p*-(1 → (6.03%). In this study, both SGCP3 and NGCP3 also contained three glycosidic bonds, D-Glc*p*-(1→, →4)-D-Glc*p*-(1→, and →4,6)-D-Glc*p*-(1 → (Table 5). The glycosidic bond →4)-α-D-Glc*p*-(1 → being connected to the main chain, and the end group, α-D-Glc*p*-(1→, being connected to the main chain through an O-6 bond. Xiang et al. (45) found that polysaccharides extracted from mussels had excellent antioxidant capacity. The structures of SGCP3 and NGCP3 found in this study are similar to those of mussel polysaccharides (main chain linkage by glycosidic bond →4)-α-D-Glc*p*-(1→, end group α-D-Glc*p*-(1 → and α-D-Glc*p*-(1 → 6)-α-D-Glc*p*-(1 → with →4,6)-α-D-Glc*p*-(1 → connected to the main chain through the O-6 bond). Because the glycosidic bonds →4,6)-α-D-Glc*p*-(1 → and α-D-Glc*p*-(1 → are the key factors affecting the polysaccharide antioxidant activity, more glycosidic bonds →4,6)-α-D-Glc*p*-(1 → and α-D-Glc*p*-(1 → were present in NGCP3 than in SGCP3 at the same polysaccharide concentration, which led to a higher antioxidant activity.

## 4 Conclusion

This study extracted SGCP and NGCPs through optimized extraction conditions and revealed significant differences in their structural characteristics and antioxidant activities. Compared to NGCPs, SGCPs exhibit significant reductions in free radical scavenging activity and protective effect against H_2_O_2_-induced cellular oxidative damage. NMR and methylation analysis further indicated that while both SGCP3 and NGCP3 have similar monosaccharide components and main-chain structure, sulfur fumigation altered their monosaccharide molar ratios and the number of repeating unit structures. Additionally, the difference in molecular weights further highlight the chemical modification effect of sulfur fumigation on GR polysaccharides. Overall, our results showed that the importance of sulfur fumigation processing technology in determining the structural and functional quality of GR polysaccharides. This suggests that sulfur fumigation adversely affects the bioactivity of these polysaccharides. Future research should focus on exploring alternative processing methods to better preserve or enhance the antioxidant properties of these valuable compounds.

## Data Availability

The original contributions presented in the study are included in the article/Supplementary material, further inquiries can be directed to the corresponding authors.
