# Supplementary material for: Comparative analysis of antioxidant activity and structural changes of Gastrodiae Rhizoma polysaccharides between sulfur-fumigation and nonsulfur-fumigation
Source: Front Nutr. 2024 Dec 4;11:1477689. doi: 10.3389/fnut.2024.1477689 (PMC11653586; doi:10.3389/fnut.2024.1477689)
Supplement: Supplementary file 1 [file Data_Sheet_1.pdf]

Supplemental methods

Chemical methods to assess antioxidant activity

1. Measurement of DPPH free radical-scavenging capacity

Weigh 4 mg of DPPH dissolved in 100 mL of anhydrous ethanol to prepare a DPPH-ethanol solution and keep it in the dark. Pipette 500 μL of different concentrations of polysaccharide, add equal volumes of deionized water and DPPH solution to them, vortex to mix, and then centrifuge after 30 min of standing at room temperature in the dark. The supernatant was collected and the absorbance was measured at 517 nm. Vc was used as the positive control, and deionized water was used as the blank control. Each sample was tested thrice independently. The DPPH radical-scavenging rate was calculated according to the following formula:

$$\text{DPPH radical scavenging rate (\%)} = (1 - \frac{A_1 - A_2}{A_0}) \times 100\%$$

A_1 is the absorbance of the mixed sample;

A_2 is the absorbance of the DPPH-free sample only (anhydrous ethanol instead of DPPH);

A_0 is the absorbance of the control (deionized water instead of sample).

2. Measurement of hydroxyl radical scavenging capacity

Polysaccharide samples (1-5 mg/mL; 500 μL) were accurately pipetted into 5-mL centrifuge tubes, and 500 μL of deionized water was added to 500 μL of the sample. Then, 500 μL of 6 mmol/L $FeSO_4$ and salicylic acid ethanol solution and 500 μL of 8 mmol/L H_2O_2 solution were added successively, followed by the measurement of the absorbance at 510 nm after heating in a water bath at 37℃ for 1 h. V was used as the positive control and deionized water as the blank control. The absorbance was measured at 510 nm after heating at 37℃ for 1 h. VC was used as the positive control, and deionized water was used as the blank control. Each sample was tested thrice independently. The hydroxyl radical-scavenging rate was calculated according to the following formula:

Hydroxyl radical scavenging rate (%) $= (1 - \frac{A_1 - A_2}{A_0}) \times 100\%$

A_1 is the absorbance of the mixed sample;

A_2 is the absorbance of the sample without H_2O_2 only (deionized water instead of H_2O_2);

A_0 is the absorbance of the control (deionized water instead of sample and H_2O_2).

3. Measurement of ABTS$^{+}\cdot$ free radical-scavenging capacity

Preparation of the ABTS$^{+}\cdot$ working solution: 6.623 mg of potassium persulfate ($K_2S_2O_8$) 6.623 mg was weighed, mixed with 10 mL of 7 mmol/L ABTS aqueous solution, stirred, and then incubated in the dark at 25°C for 12-16 h to form a blue-green solution. The solution was then diluted (about 75-fold) with PBS at a concentration of 1 μmol/L (pH 7.4), and the absorbance was read at 734 nm. The absorbance of the ABTS$^{+}\cdot$ working solution minus that of the blank control PBS was expected to be 0.70 ±0.03. Sample assay: polysaccharide samples with different concentrations of 1-5 mg/mL were configured, and 0.01 mL of the sample was taken; 0.2 mL of ABTS^{+}-Vc was used as positive control and deionized water was used as blank control. Each sample was independently tested thrice. ABTS$^{+}\cdot$ radical-scavenging rate was expressed as follows:

$$\text{ABTS}^{+}\cdot \text{ radical scavenging rate (\%)} = (1 - \frac{A_1 - A_2}{A_0}) \times 100\%$$

A_1 is the absorbance value of the mixed sample;

A_2 is the absorbance value of the sample without ABTS$^{+}\cdot$ working solution (PBS instead of ABTS$^{+}\cdot$ working solution);

A_0 is the absorbance value of the control (deionized water instead of sample).

4. Determination of ferrous ion chelating capacity

Deionized water (0.5 mL) was mixed with 12.5 μL of 2 mmol/L $FeCl_2$ solution, and 100 μL of polysaccharide solutions of different concentrations (1-5 mg/L) were added. The mixed solution was kept at 25°C for 3 min, and then 25 μL of phenanthrozine solution (5 mmol/L) was added. Subsequently, the mixture was

shaken and incubated for 10 min at 25°C. The absorbance of the mixture was measured at 562 nm using an enzyme meter, with disodium ethylenediaminetetraacetic acid (EDTA-2Na) solution as a positive control and deionized water as a blank control. The ferrous ion chelating capacity was calculated as follows:

$$\text{Ferrous ion-chelating capacity (\%)} = (1 - \frac{A_1 - A_2}{A_0}) \times 100\%$$

A_1 is the absorbance value of the sample solution;

A_2 is the absorbance value of the sample without $FeCl_2$ only (deionized water instead of $FeCl_2$);

A_0 is the absorbance value of the control (deionized water instead of sample).

5. Determination of total reducing power

Pipette 500 μL of 1-5 mg/mL polysaccharide samples, to which 500 μL of potassium ferricyanide solution and 500 μL of phosphate buffer solution (pH 6.6, 0.2 mol/L) was added and mixed thoroughly, followed by heating it in a water bath at 50℃ for 20 min. Then, 500 μL of 10% TFA was added and the two were mixed thoroughly, followed by centrifugation (3000 rpm/min, 10 min); of the supernatant, 1 mL was mixed with 1 mL of ferric chloride solution containing 0.1% TFA and the absorbance was measured with a UV spectrophotometer at 700 nm. The measured absorbance indicates the total reducing power. Each sample was tested independently three times. The total reducing power curve was plotted with polysaccharide concentration as the horizontal coordinate and the absorbance value as the vertical coordinate.
