# Supplementary material for: Comparative analysis of antioxidant activity and structural changes of Gastrodiae Rhizoma polysaccharides between sulfur-fumigation and nonsulfur-fumigation
Source: Front Nutr. 2024 Dec 4;11:1477689. doi: 10.3389/fnut.2024.1477689 (PMC11653586; doi:10.3389/fnut.2024.1477689)
Supplement: Supplementary file 2 [file Image_1.pdf]

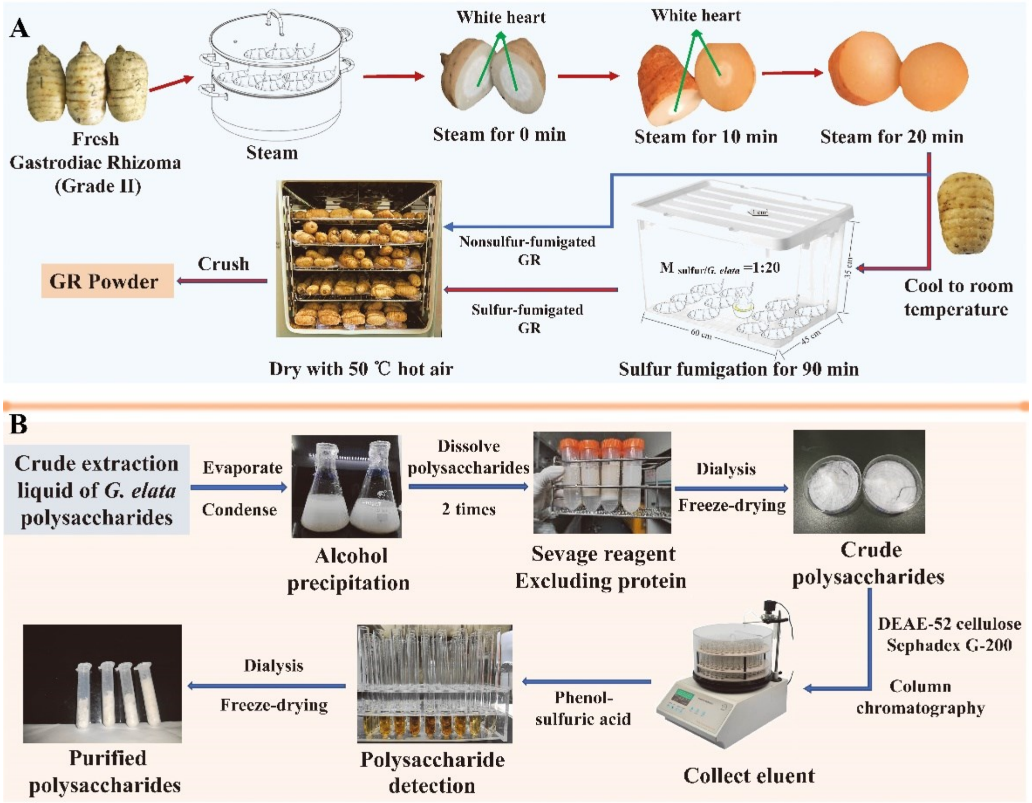

Supplemental Fig. 1S Fumigation process of *Gastrodiae Rhizoma* samples (A) and separation and purification process of *Gastrodiae Rhizoma* crude polysaccharides (B).

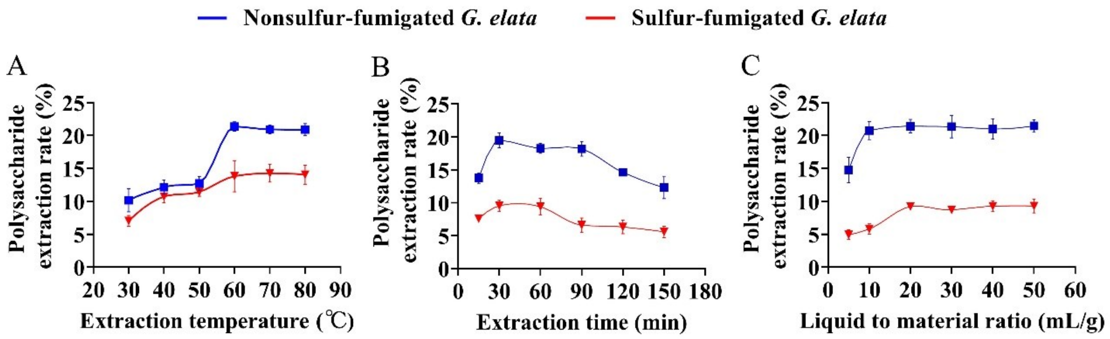

Supplemental Fig. 2S Effect of (A) extraction temperature, (B) extraction time, and (C) liquid/feed ratio on the extraction rate of crude polysaccharides of sulfurized and unsulfurized aspens.

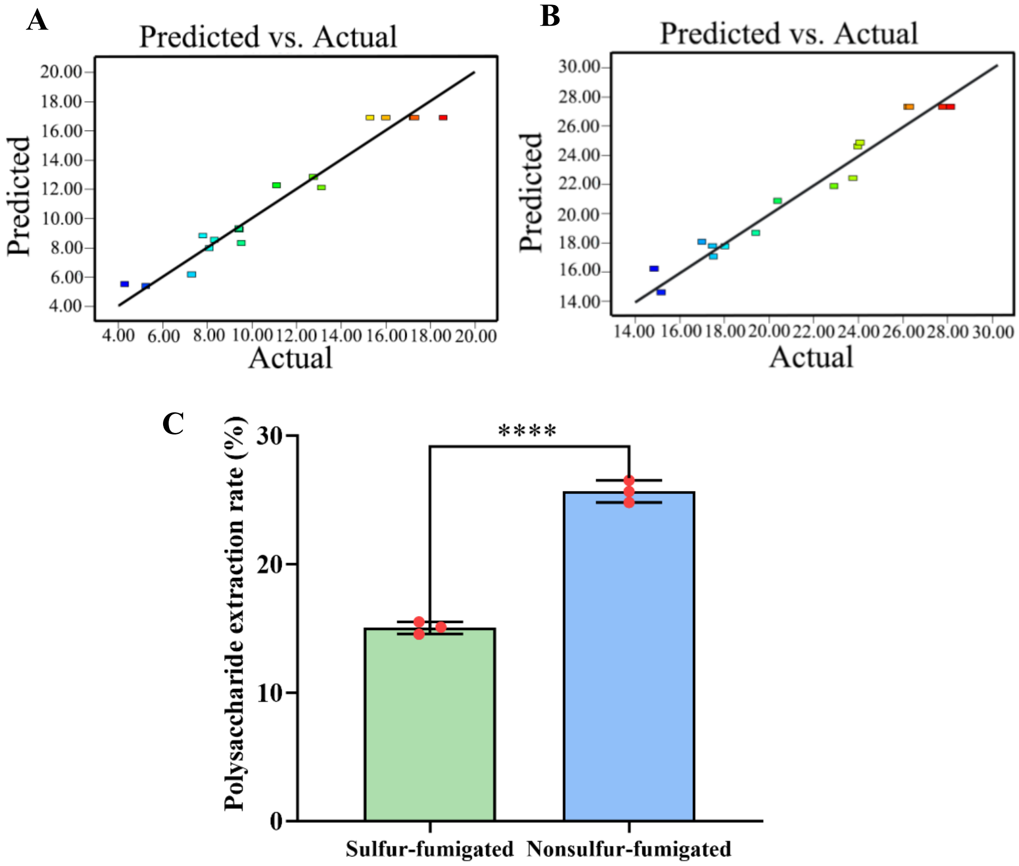

Supplemental Fig. 3S The model predicted value and the experimental actual value of sulfur-fumigated (A) and nonsulfur-fumigated (B) *Gastrodiae Rhizoma* crude polysaccharides. Actual extraction rates of sulfur-fumigated and nonsulfur-fumigated *Gastrodiae Rhizoma* crude polysaccharides under optimal extraction process conditions (C).

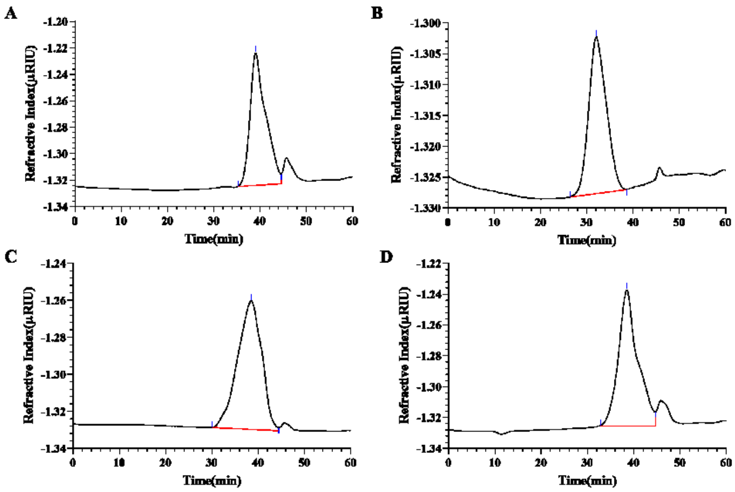

Supplemental Fig. 4S HPGPC chromatogram. The peak with a retention time of 45.6 min is the peak of the mobile phase. (A) SGCP2; (B) SGCP3; (C) NGCP2; (D) NGCP3.

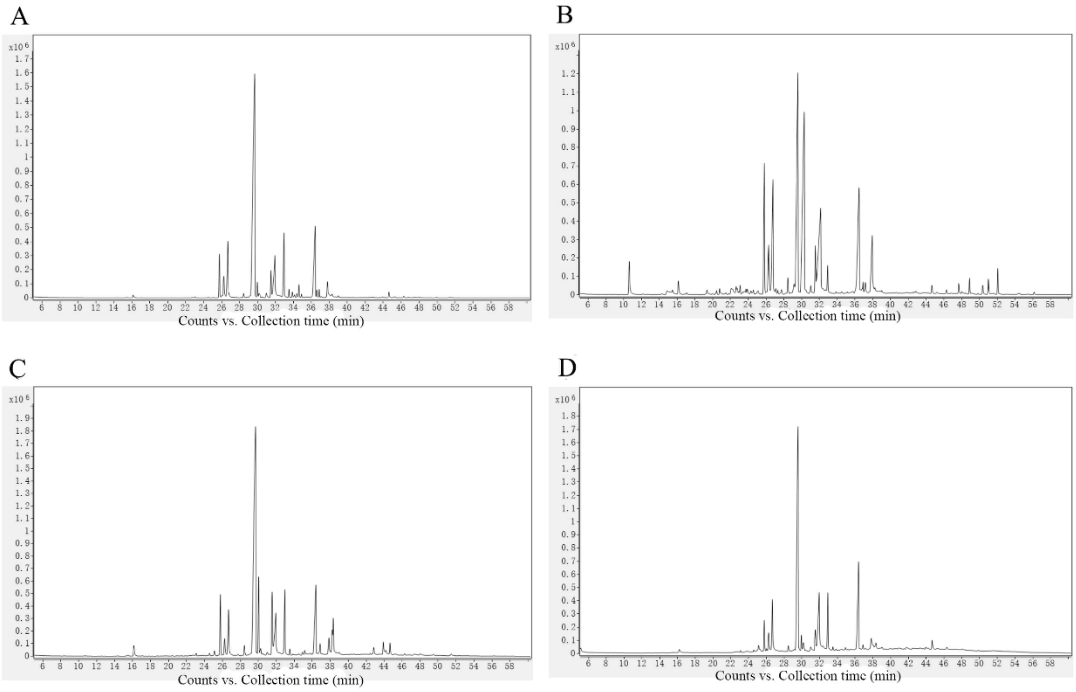

Supplemental Fig. 5S GC-MS total ion flow diagrams of PMAAs belonging to sulfur-fumigated *G. elata* polysaccharides (A) SGCP2, (B) SGCP3, nonsulfur-fumigated *Gastrodiae Rhizoma* polysaccharides (C) NGCP2, (D) NGCP3

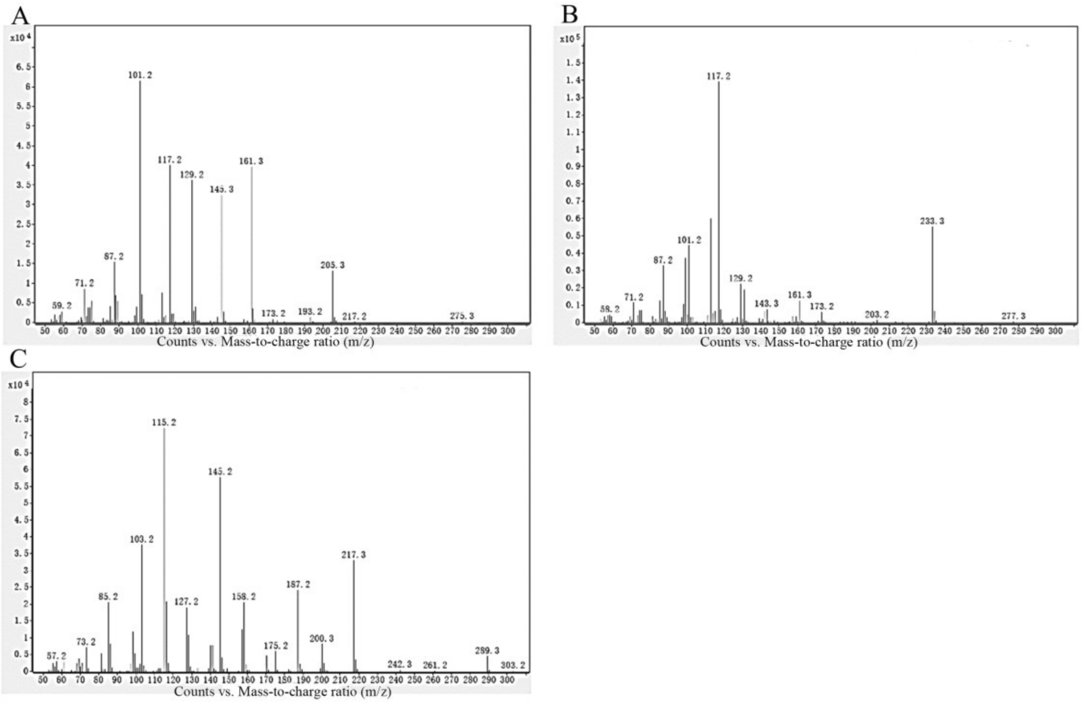

Supplemental Fig. 6S Mass spectra of each PMAAs belonging to SGCP2 (A) 2,3,4,6-Me_4-Glc*p*, (B) 2,3,6-Me_3-Glc*p,* and (C) 2,3-Me_2-Glc*p*

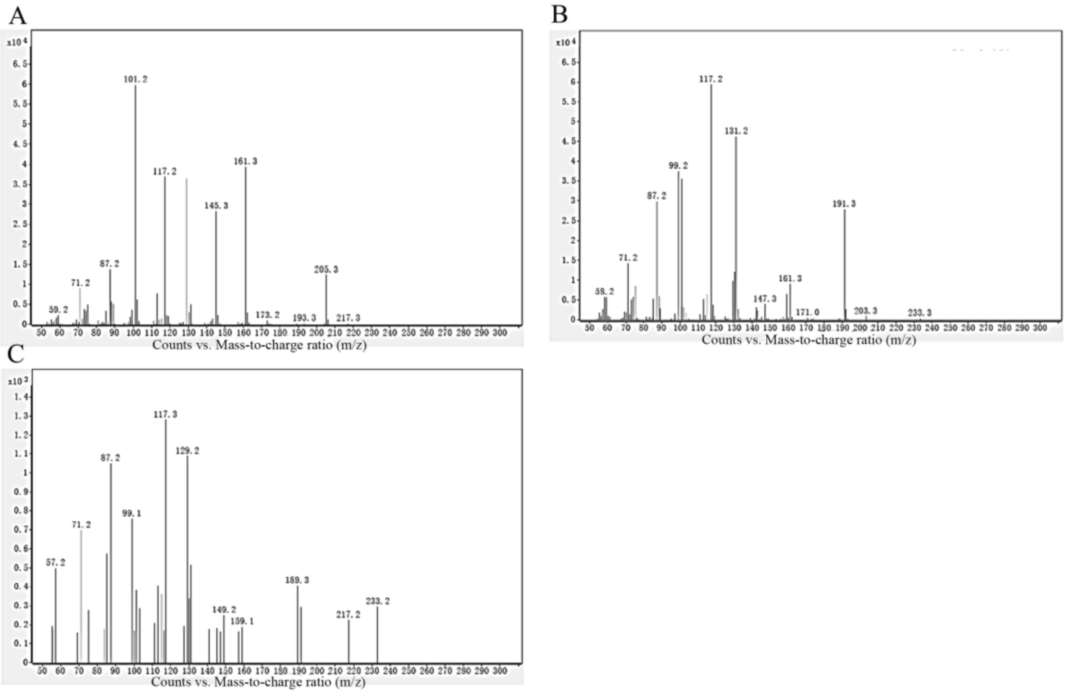

Supplemental Fig. 7S Mass spectra of each PMAA belonging to SGCP3. (A) 2,3,4,6-Me_4-Glc*p*, (B) 2,3,6-Me_3-Glc*p*, (C) 2,3-Me_2-Glc*p*

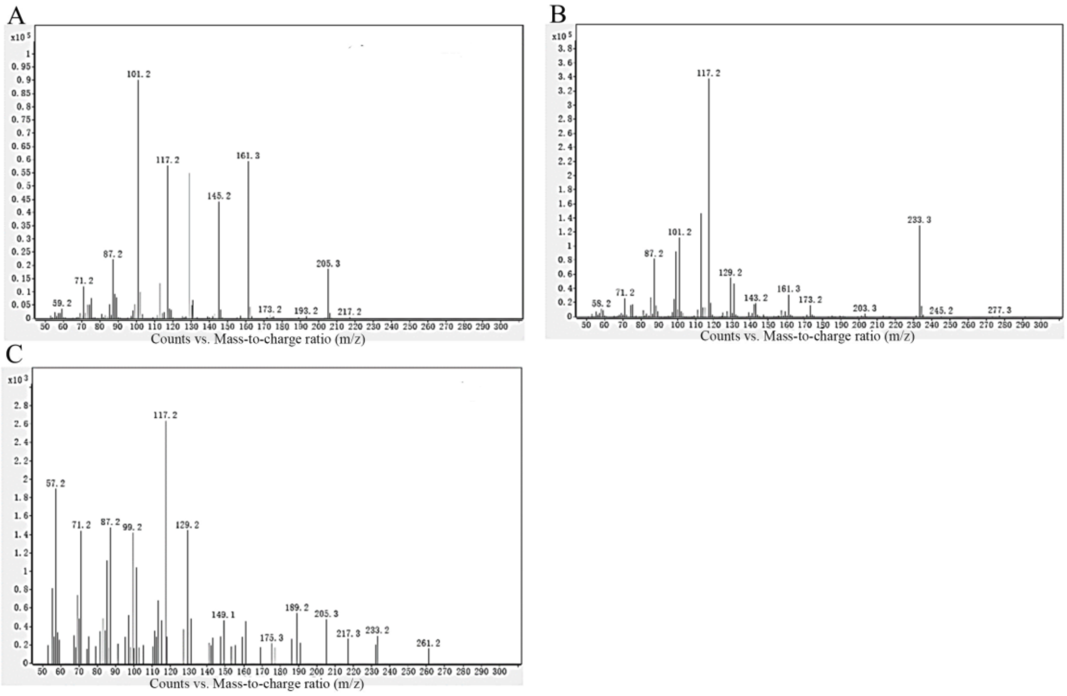

Supplemental Fig. 8S Mass spectra of each PMAA belonging to NGCP2. (A) 2,3,4,6-Me_4-Glc*p*, (B) 2,3,6-Me_3-Glc*p,* and (C) 2,3-Me_2-Glc*p*

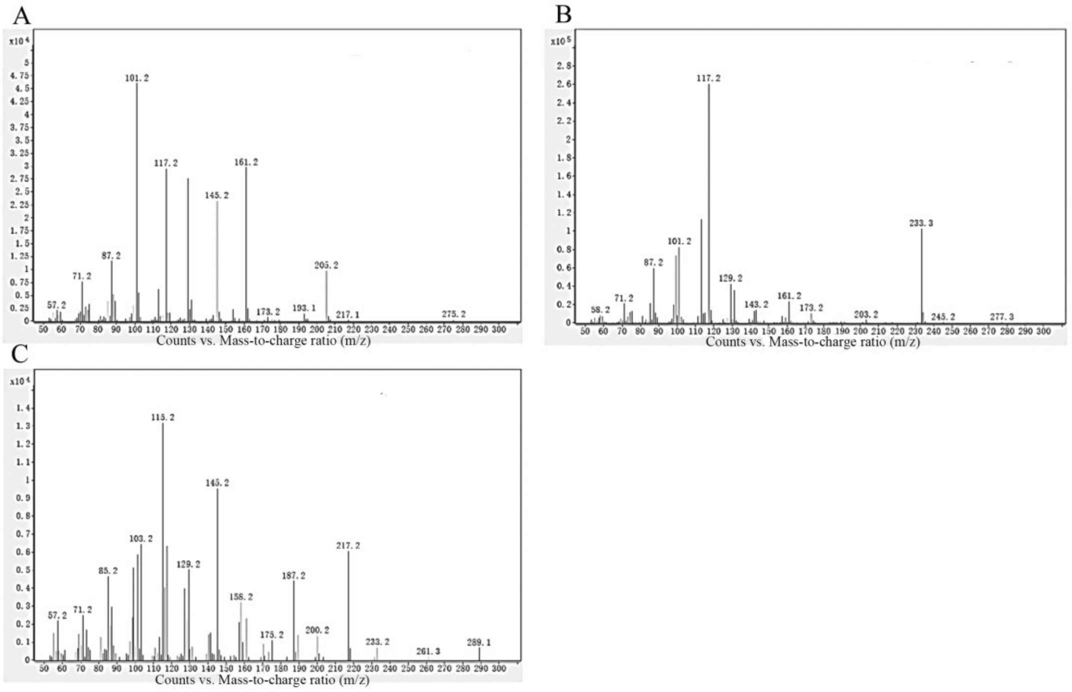

Supplemental Fig. 9S Mass spectra of each PMAA belonging to NGCP2. (A) 2,3,4,6-Me_4-Glc*p*, (B) 2,3,6-Me_3-Glc*p*, (C) 2,3-Me_2-Glc*p*
