# Supplementary material for: Comparative analysis of antioxidant activity and structural changes of Gastrodiae Rhizoma polysaccharides between sulfur-fumigation and nonsulfur-fumigation
Source: Front Nutr. 2024 Dec 4;11:1477689. doi: 10.3389/fnut.2024.1477689 (PMC11653586; doi:10.3389/fnut.2024.1477689)
Supplement: Supplementary file 3 [file Table_1.docx]

Supplemental Table 1S Standard curve regression equation.

| Monosaccharide | Standard curve equation | R^2^ |
| --- | --- | --- |
| Man | y = 1*10^15^x - 73089 | 0.9994 |
| Rha | y = 6*10^14^x - 18639 | 0.9991 |
| GlcA | y = 3*10^14^x - 551774 | 0.9995 |
| GalA | y = 7*10^14^x - 168076 | 0.9998 |
| Glc | y = 3*10^15^x + 3000000 | 0.9996 |
| Gal | y = 1*10^15^x - 39752 | 1 |
| Xyl | y = 3*10^14^x + 94671 | 0.9998 |
| Ara | y = 7*10^14^x - 15018 | 1 |

Note: X is the molar mass, and y is the peak area.

Supplemental Table 2S ^1^H NMR and ^13^C NMR chemical shift attribution of monosaccharide residues in *Gastrodiae Rhizoma* polysaccharides.

| Polysaccharides | Glycosidic bond | H1/C1 | H2/C2 | H3/C3 | H4/C4 | H5/C5 | H6a /C6 | H6b |
| --- | --- | --- | --- | --- | --- | --- | --- | --- |
| SGCP3 | Residue A  →4)-α-D-Glc*p*-(1→ | 5.22 | 3.43 | 3.78 | 3.49 | 3.64 | 3.58 | 3.66 |
|  |  | 100.57 | 72.82 | 74.19 | 77.55 | 72.44 | 61.29 |  |
|  | Residue B  →4,6)-α-D-Glc*p*-(1→ | 4.79 | 3.4 | 3.53 | 3.65 | 3.22 | 3.69 | 3.8 |
|  |  | 99.8 | 72.77 | 73.93 | 77.41 | 70.6 | 68.37 |  |
|  | Residue C  α-D-Glc*p*-1→ | 5.17 | 3.51 | 3.61 | 3.86 | 3.88 | 3.55 | 3.71 |
|  |  | 101.24 | 71.85 | 73.94 | 70.52 | 71.59 | 62.21 |  |
| NGCP3 | Residue A  →4)-α-D-Glc*p*-(1→ | 5.23 | 3.47 | 3.78 | 3.48 | 3.7 | 3.62 | 3.59 |
|  |  | 100.51 | 72.83 | 74.48 | 77.50 | 72.45 | 61.81 |  |
|  | Residue B  →4,6)-α-D-Glc*p*-(1→ | 4.80 | 3.41 | 3.53 | 3.45 | 3.22 | 3.7 | 3.81 |
|  |  | 99.33 | 72.78 | 73.94 | 77.02 | 70.61 | 68.38 |  |
|  | Residue C  α-D-Glc*p*-1→ | 5.18 | 3.52 | 3.62 | 3.87 | 3.89 | 3.62 | 3.59 |
|  |  | 101.25 | 71.86 | 73.95 | 70.53 | 71.6 | 61.2 |  |
